# Identification of Clinical Phenotypes in Septic Patients Presenting With Hypotension or Elevated Lactate

**DOI:** 10.3389/fmed.2022.794423

**Published:** 2022-05-19

**Authors:** Zachary T. Aldewereld, Li Ang Zhang, Alisa Urbano, Robert S. Parker, David Swigon, Ipsita Banerjee, Hernando Gómez, Gilles Clermont

**Affiliations:** ^1^UPMC Children's Hospital of Pittsburgh, Pittsburgh, PA, United States; ^2^Department of Critical Care Medicine, University of Pittsburgh, Pittsburgh, PA, United States; ^3^Department of Pediatrics, University of Pittsburgh, Pittsburgh, PA, United States; ^4^Department of Chemical and Petroleum Engineering, Swanson School of Engineering, University of Pittsburgh, Pittsburgh, PA, United States; ^5^Department of Mathematics, University of Pittsburgh, Pittsburgh, PA, United States

**Keywords:** septic shock, phenotypes, machine learning, sepsis, hierarchical clustering

## Abstract

**Introduction:**

Targeted therapies for sepsis have failed to show benefit due to high variability among subjects. We sought to demonstrate different phenotypes of septic shock based solely on clinical features and show that these relate to outcome.

**Methods:**

A retrospective analysis was performed of a 1,023-subject cohort with early septic shock from the ProCESS trial. Twenty-three clinical variables at baseline were analyzed using hierarchical clustering, with consensus clustering used to identify and validate the ideal number of clusters in a derivation cohort of 642 subjects from 20 hospitals. Clusters were visualized using heatmaps over 0, 6, 24, and 72 h. Clinical outcomes were 14-day all-cause mortality and organ failure pattern. Cluster robustness was confirmed in a validation cohort of 381 subjects from 11 hospitals.

**Results:**

Five phenotypes were identified, each with unique organ failure patterns that persisted in time. By enrollment criteria, all patients had shock. The two high-risk phenotypes were characterized by distinct multi-organ failure patterns and cytokine signatures, with the highest mortality group characterized most notably by liver dysfunction and coagulopathy while the other group exhibited primarily respiratory failure, neurologic dysfunction, and renal dysfunction. The moderate risk phenotype was that of respiratory failure, while low-risk phenotypes did not have a high degree of additional organ failure.

**Conclusions:**

Sepsis phenotypes with distinct biochemical abnormalities may be identified by clinical characteristics alone and likely provide an opportunity for early clinical actionability and prognosis.

## Introduction

Sepsis is a syndrome caused by a dysregulated systemic response to infection resulting in organ dysfunction (1). Clinical features vary among patients depending on site of infection, patient characteristics, and time between onset of infection and presentation (2). Likely due to this heterogeneity as well as our poor understanding of the mechanisms at play, sepsis-targeted treatments and strategies that showed promise in pre-clinical settings have largely been unsuccessful in humans (2–8). Increasing evidence suggests that some of this lack of efficacy may be due to applying a one-size-fits-all approach to patients with sepsis. Phenotypic differences are observed, and secondary analyses show subgroups of patients that do benefit from specific interventions (9). Similar work has been performed in ARDS, demonstrating benefit to a high PEEP strategy only in a hyperinflammatory phenotype (10). Thus, there is growing interest in a more tailored approach to sepsis treatment, but the best means of delivering this has not yet been determined (11).

The first step is to identify subgroups of patients expressing phenotypic similarity. An ideal means of accomplishing this would rely on clinical variables that can be obtained early in a patient's presentation, thus relying on a combination of vital signs and expedient laboratory measurements. This would allow early classification of patients for randomization to study treatment arms so that targeted therapies could be trialed. Theoretically, phenotypic similarity could be due to similar underlying pathophysiology, and therefore could suggest treatment targets. A recently published electronic health record (EHR)-based study identified four broad phenotypes of patients, one of which had much higher mortality (12). Focusing on patients with proven early septic shock, we extend this work to explore variations within this group. We hypothesized that these phenotypes would correspond to underlying biochemical differences and evolve differently in time, and that most importantly, they might provide therapeutically actionable targets. Using a cohort of patients with a proven septic shock diagnosis from the Protocol-Based Care for Early Septic Shock (ProCESS) trial, which offers a rich dataset of clinical data as well as some measurement of plasma markers, we construct clinical phenotypes using agglomerative hierarchical clustering techniques (13). Ultimately, we found that the types we defined had distinct clinical features, including mortality, patterns of organ failure, and need for organ system support.

## Methods

A retrospective analysis of the ProCESS trial was conducted. This trial enrolled 1,341 subjects into one of three treatment arms to test the efficacy of early goal directed therapy in early septic shock. Treatment arms were not taken into consideration for clustering analysis because the ProCESS investigators concluded that there were no statistically significant differences in patient outcomes between the three arms of the trial. Clusters were identified using baseline clinical characteristics, prior to the potential impact of any trial intervention.

Eighty-four clinical variables were measured at baseline (time of enrollment to the ProCESS trial), 6, 24, and 72 h. To identify clusters in a clinically actionable way, we elected to define phenotypes using only variables that are regularly obtained such as vital signs and routine labs. Laboratory studies that are not routinely measured, such as cytokines, were not included in the clustering analysis. For markers that demonstrated a high degree of correlation, the marker with fewer measurements was eliminated. To incorporate the degree of cardiovascular and respiratory support needed, a cardiovascular score and respiratory score were devised. Cardiovascular score was based on cardiac SOFA score with slight modification (see Table 1); norepinephrine was the most commonly used vasopressor. Due to a paucity of oxygenation information, a similar adaptation could not be made for the respiratory score. Thus, we defined respiratory score as follows: 0 indicated no mechanical ventilation (MV); 1 indicated non-invasive ventilation (CPAP or BiPAP); 2 indicated invasive MV with PEEP ≤ 5 cmH_2_O; 3 indicated invasive MV with PEEP > 5 cmH_2_O.

**Table 1:** CV score criteria based on cardiac SOFA score.

**CV Score**	**Criteria**
0	mean arterial pressure (MAP) ≥ 70 mmHg without pressor support
1	MAP < 70 mmHg without pressor support
2	dopamine ≤ 5 mcg/kg/min or dobutamine (any dose) or phenylephrine (any dose)
3	15 mcg/kg/min ≥ dopamine > 5 mcg/kg/min but, or total dose of epinephrine and norepinephrine ≤ 0.1 mcg/kg/min
4	dopamine > 15 mcg/kg/min or total dose of epinephrine and norepinephrine > 0.1 mcg/kg/min

To increase variable availability, absent baseline values were substituted by a measurement closest, within 3 h of enrollment time. To identify study cohort of maximum size, we first identified several edge-maximum bicliques in the dataset containing at least 20 variables, where an edge represents a specific patient with a specific variable measurement (14, 15). We then augmented the number of markers as to preserve <20% missingness in any given variable. This yielded a total of 1,023 of the original 1,341 subjects. When missing, variables were imputed using a predictive mean matching algorithm from the MICE (Multivariate Imputation by Chained Equations) package in R. We confirmed univariate statistical identity between original and imputed data. A given subject's variable value was ranked using this variable's empirical cumulative distribution function constructed from the baseline values from all patients. Centile of values were linearly mapped to a range of −10 to 10. Thus, a value of zero represents the population median for a variable, but may not be in the “normal” range.

### Phenotype Identification and Analysis

Hierarchical clustering was selected as the underlying clustering algorithm. To create derivation and validation cohorts, study centers were randomized to each in a roughly 2 to 1 ratio. In order to remove much of the subjectivity typically involved in determining cluster number in hierarchical clustering, consensus clustering using the ConsensusClusterPlus package in R was used to optimize cluster number on the baseline values. We refer the reader to Wilkerson (16) for a full explanation of consensus clustering. Briefly, consensus clustering aims to identify the ideal number of clusters and corresponding memberships in a dataset by repeated subsampling of the dataset and subsequent clustering of the subsamples. Pairwise consensus values are obtained that give the proportion of times two subjects were in the same cluster out of the number of times they were taken together in a subsample. This is expressed graphically in consensus plots, and graphs with a high amount of intra-cluster consensus and low amount of inter-cluster consensus are indicative of better results. In combination with a cumulative distribution function as well as graphical observation of stable or unstable clusters, an ideal number of clusters can be chosen, which also yields cluster membership. Thus, this technique provides a means of validation for both cluster number and cluster membership. For our implementation, we chose 80% subsampling of subjects with 1,000 resamplings, along with the Euclidean distance metric and the Ward D2 linkage. Other distance metrics, methods of agglomeration, and clustering methods were trialed but generally yielded a similar or greater degree of inter-cluster consensus, and thus less interpretable results. Clustering was performed after rescaling each variable according to ranks as discussed above. We believe this ranking approach allows increased discrimination between measurements, as other approaches, such as standardization, affords little discrimination for variables that are non-normally distributed, especially if there are a significant number of outliers. We included comparable number of variables representing different organ systems, to balance the contribution of each system to the attribution of similarity.

As discussed above, clusters were identified using only the baseline values. Behavior of clusters was then examined over time within the confines of the original cluster memberships. The clustering analysis was first performed on the derivation cohort and then repeated on the validation cohort. To compare variables across phenotypes, we performed analysis-of-variance (ANOVA) tests for continuous variables and chi-square tests for categorical variables. For variables that were found to be significantly different, pairwise comparisons were performed using the Tukey test. Kaplan-Meier survival analysis to 60 and 365 days was performed and differences among phenotypes types were expressed using log-rank statistic. Subjects were also dichotomized as per volume of fluid received in total in the first 24 h. The association between fluid volume and mortality across phenotypes was examined by Kaplan-Meier survival. Significance was assessed by log-rank statistic.

### Multinomial Model Development

To devise a means of prospectively identifying types with the fewest variables necessary, a multinomial model was developed using only clinical variables available at baseline. In order to help identify the most high-impact variables, preliminary models were developed using the glmnet package in R, which is an implementation of general linearized models. Briefly, this package iteratively determines the best fit model for a given number of variables out of the total number provided, and also determines the number of variables at which the best fit is achieved. However, while the same number of variables available per predicted class (phenotype) is the same, the variables selected for each class may be different. Thus, in order to determine a truly parsimonious model that relies on the same variables for all five types, the results of the glmnet model were used to inform variable selection by focusing on the highest impact variables across types. Specifically, glmnet was used to generate a multinomial model. This results in individual models for each type, which can in turn provide an associated likelihood of type membership. However, variable selection for each type is individualized, with those chosen being different for each type. For instance, in the application to the derivation cohort, the models for types L1, L2, M, H1, and H2 used 15, 13, 17, 16, and 19 variables, respectively, with only partial overlap. Thus, the impact of each variable was examined in combination with its coefficient to understand which variables exerted the greatest influence. Variables of high impact were added successively to the model until little improvement was seen, resulting in a more parsimonious model to assign cluster membership at baseline.

## Results

Subject demographics and baseline characteristics were mostly similar between the derivation and validation cohorts with a few exceptions (Table 2). Infectious source was similar between the groups. Subjects in the derivation cohort had slightly higher APACHE II scores and slightly lower liver and cardiac SOFA scores. Lactate levels were also higher in the derivation cohort. Development of new respiratory failure was significantly more common in the derivation cohort. When Sepsis-3 criteria were applied to the cohorts, 630 (98%) subjects in the derivation cohort and 370 (97%) in the validation cohort met sepsis criteria. Of those in whom values were available at 6 h, 76% in the derivation cohort and 69% in the validation cohort met Sepsis-3 septic shock criteria.

**Table 2:** Demographic and clinical comparison between cohorts.

	**Derivation** ***N*** **= 642**	**Validation** ***N*** **= 381**	***P*** **-value**
Age	60.9 ± 16.1	61.8 ± 15.9	0.3808
Sex– no. (%)	375 (58.4%)	208 (54.6%)	0.2597
Race– no. (%)			
White	424 (66.0%)	281 (73.8%)	0.0122
Black	168 (26.2%)	77 (20.2%)	0.0372
Other	50 (7.8%)	23 (6.0%)	0.3542
**Infectious source– no. (%)**			
Pneumonia	200 (31.2%)	123 (32.3%)	0.7591
Intraabdominal infection	93 (14.5%)	53 (13.9%)	0.8714
Urosepsis	138 (21.5%)	81 (21.3%)	0.9921
Skin or soft tissue infection	48 (7.5%)	27 (7.1%)	0.9145
CNS	6 (0.9%)	2 (0.5%)	0.7248
Endocarditis	0 (0.0%)	5 (1.3%)	0.0144
Catheter-related infection	21 (3.3%)	9 (2.4%)	0.5213
Unknown	78 (12.1%)	45 (11.8%)	0.9509
Other	44 (6.9%)	24 (6.3%)	0.8303
None	14 (2.2%)	12 (3.1%)	0.4554
Blood culture positive–no. (%)	204 (31.8%)	115 (30.2%)	0.6444
**Illness severity**			
APACHE II	21.7 ± 7.9	20.3 ± 7.5	0.0044
APACHE III	63.6 ± 23.5	63.3 ± 20.8	0.8725
Charleson	2.6 ± 2.6	2.7 ± 2.6	0.7043
SOFA	2.0 ± 1.6	2.4 ± 1.6	0
SOFA cardiac	0.9 ± 1.3	0.7 ± 1.1	0.0013
SOFA CNS	0.6 ± 1.0	0.5 ± 1.0	0.103
SOFA coag	0.7 ± 1.0	0.6 ± 0.9	0.1339
SOFA liver	1.5 ± 1.3	1.3 ± 1.2	0.0033
SOFA renal	1.8 ± 1.2	1.8 ± 1.2	0.6292
SOFA respiratory	7.5 ± 3.6	7.3 ± 3.6	0.2426
**Physiologic variables**			
SBP (mmHg)	98.9 ± 30.2	101.3 ± 26.3	0.1856
Heart Rate	112.0 ± 24.0	109.6 ± 23.7	0.1234
Temperature (Celsius)	37.4 ± 1.6	37.3 ± 1.4	0.4886
Respiratory rate	22.9 ± 7.2	22.0 ± 6.1	0.0392
Total bilirubin	1.5 ± 2.0	1.5 ± 2.4	0.7136
Lactate	3.4 ± 3.3	2.4 ± 1.9	0.0003
**Mortality–no. (%)**			
14 days	130 (20.2%)	61 (16.0%)	0.1098
28 days	154 (24.0%)	80 (21.0%)	0.3059
60 days	182 (28.3%)	99 (26.0%)	0.4552
1 year	234 (36.4%)	135 (35.4%)	0.7951
Multiorgan failure, baseline	384 (59.8%)	216 (56.7%)	0.3607
**New organ failure–no. (%)**			
Cardiac	401 (62.5%)	247 (64.8%)	0.4884
Renal	30 (4.7%)	9 (2.4%)	0.0897
Respiratory	254 (39.6%)	122 (32.0%)	0.0187
Hosp LOS	11.7 ± 10.9	11.2 ± 10.4	0.4804
ICU LOS	4.9 ± 6.0	5.1 ± 7.1	0.6555
Number of SAEs	15 (2.3%)	10 (2.6%)	0.2571
**Subject disposition category– no. (%)**			
Home	323 (50.3%)	200 (52.5%)	0.5417
SNF	119 (18.5%)	62 (16.3%)	0.4053
Dead	146 (22.7%)	77 (20.2%)	0.3844

### Determination of Clusters

Five clusters were selected as the optimal number of clusters primarily based on inspection of the consensus matrices, cumulative distribution functions, and tracking plot that follows a subject's cluster membership over varying numbers of clusters (Supplementary Figures 1–3, respectively). In the consensus matrices of Supplementary Figure 1, subjects are ordered symmetrically along the x and y axes. Dark blue indicates that two subjects were in the same cluster a high percentage of resamplings in which both were selected. White indicates that they were never in the same cluster. Thus, results that have clusters that are dark blue with little to no blue outside of the cluster are indicative of high intra-cluster consensus and low inter-cluster consensus, respectively. As seen in the plots, 5 clusters result in much less inter-cluster consensus than 4 clusters.

In Supplementary Figure S2, plot A shows the cumulative distribution function for varying number of clusters, while plot B demonstrates the change in the area under these curves. There is clear improvement as cluster number is increased from 2 to 3 and from 3 to 4. Based on plot B, the improvement going from 4 to 5 is certainly greater than subsequent increases, though not as great as that seen for 3 to 4.

The plot shown in Supplementary Figure S3 tracks cluster membership over varying numbers of clusters and thus allows another way of judging stability of clusters. Subjects are arranged along the x-axis, and cluster number increases down the y-axis. As can be seen, each successive increase in cluster number up to 5 results in the formation of a stable new, large cluster. After 5 clusters, there is primarily the formation of “sliver” clusters, which argue against 6 or more clusters. Examination of the result of subdividing 4 clusters into 5 resulted primarily in the formation of a group with the most minimal organ dysfunction of any group, and thus supported the notion that this is indeed a distinct group. Thus, based on the consensus plot of Supplementary Figure S1 and tracking plot of Supplementary Figure S3, the decision was made to select 5 clusters rather than 4.

**Figure 1:**
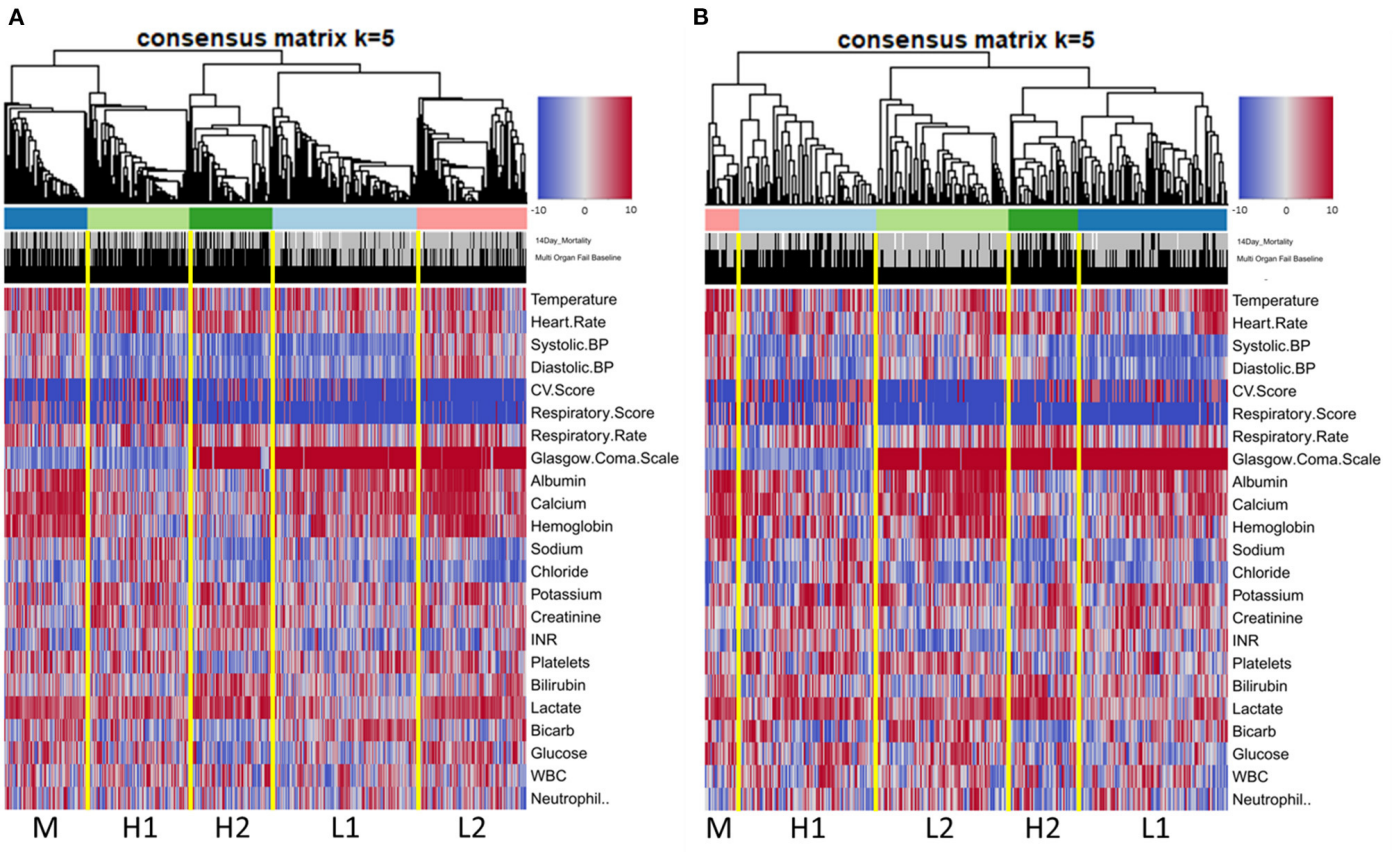
Heatmap of ranked clinical variables by phenotype at baseline in derivation cohort **(A)** and validation cohort **(B)**.

Clinical characteristics of these five groups are displayed graphically in the heatmap in Figure 1A. Inspection reveals distinct patterns of organ dysfunction further correlated in Table 3 and discussed below. Of note, we did not adjust for multiple comparisons, and thus conclusions should be considered exploratory.

**Table 3:** Comparison of clustered clinical variables and plasma markers across phenotypes at baseline in the derivation cohort. Phenotypes H1 and H2 were specifically compared against each other using Tukey tests because these were the two highest mortality groups.

	**L1** ***n*** **= 178 (28%)**	**L2** ***n*** **= 135 (21%)**	**M** ***n*** **= 102 (16%)**	**H1** ***n*** **= 125 (19%)**	**H2** ***n*** **= 102 (16%)**	***P*** **-value**	**H1 vs H2**
**Clustered clinical variables, mean ±SD**							
Temperature	37.64 (1.26)	37.64 (1.18)	38.03 (1.43)	36.87 (1.72)	37.23 (1.35)	<0.0001	0.2976
Heart rate	102.15 (17.68)	107.53 (21.04)	112.15 (18.67)	103.70 (26.80)	116.33 (17.93)	<0.0001	0.0001
Systolic BP	82.56 (11.43)	113.74 (24.79)	107.14 (21.83)	85.39 (15.11)	84.04 (17.95)	<0.0001	0.9816
Diastolic BP	46.64 (8.87)	65.03 (14.47)	59.11 (14.55)	45.20 (11.99)	44.96 (10.84)	<0.0001	0.9999
CV score^*^	0.93 (1.39)	0.15 (0.70)	0.75 (1.41)	1.70 (1.68)	1.04 (1.45)	<0.0001	0.0028
Respiratory score	0.08 (0.31)	0.15 (0.54)	0.82 (1.01)	0.93 (1.11)	0.22 (0.59)	<0.0001	<0.0001
Respiratory rate	23.67 (6.08)	26.75 (8.33)	25.12 (7.83)	24.52 (8.66)	26.80 (7.12)	0.0011	0.1588
Glasgow Coma Scale	14.98 (0.19)	14.92 (0.30)	9.73 (4.19)	9.83 (4.10)	14.51 (1.60)	<0.0001	<0.0001
Albumin	3.08 (0.77)	3.62 (0.89)	3.38 (0.75)	2.42 (0.79)	2.50 (0.75)	<0.0001	0.9569
Calcium	8.58 (1.04)	9.02 (1.11)	9.14 (1.02)	8.00 (0.92)	7.94 (0.94)	<0.0001	0.9909
Hemoglobin	11.36 (2.31)	12.83 (2.71)	13.30 (2.11)	10.20 (2.17)	9.89 (2.60)	<0.0001	0.8754
Sodium	135.64 (5.41)	134.84 (6.32)	139.23 (4.96)	139.73 (8.45)	132.85 (5.90)	<0.0001	<0.0001
Chloride	101.04 (7.06)	96.81 (8.22)	101.75 (7.27)	105.63 (9.40)	99.51 (7.37)	<0.0001	<0.0001
Potassium	4.11 (0.80)	4.45 (1.05)	4.22 (0.90)	4.58 (1.19)	4.65 (1.14)	<0.0001	0.9853
Creatinine	1.98 (1.67)	2.54 (2.36)	1.88 (1.46)	3.02 (2.13)	3.33 (2.52)	<0.0001	0.7928
INR	1.58 (1.91)	1.97 (1.74)	1.66 (1.04)	2.05 (1.87)	2.25 (1.61)	0.0551	0.9558
Platelets	229.79 (129.77)	230.45 (138.04)	257.95 (137.86)	232.51 (151.16)	136.57 (108.28)	<0.0001	<0.0001
Total bilirubin	1.33 (2.47)	1.75 (2.59)	1.04 (0.79)	1.24 (2.62)	3.25 (3.68)	<0.0001	<0.0001
Lactate	3.09 (1.98)	6.09 (2.78)	5.91 (2.57)	5.21 (3.78)	7.56 (4.93)	<0.0001	<0.0001
Bicarb	23.28 (4.25)	19.45 (5.66)	22.39 (6.24)	19.69 (7.07)	17.66 (4.26)	<0.0001	0.1152
Glucose	140.25 (80.28)	235.64 (210.49)	217.87 (184.62)	186.63 (170.34)	128.94 (89.62)	<0.0001	0.041
WBC count	14.85 (9.25)	16.56 (9.54)	16.36 (11.31)	18.13 (14.70)	18.55 (14.71)	0.0682	0.9989
Neutrophil %	75.78 (20.23)	73.17 (23.69)	75.71 (21.81)	73.69 (22.09)	68.32 (25.87)	0.1122	0.4334
**Plasma markers, median (IQR)**							
TNF	28.00 (17.41–32.61)	28.00 (14.73–30.48)	28.00 (16.67–35.01)	28.00 (20.49–32.74)	28.00 (26.54–137.65)	0.0556	0.1292
IL6	164.80 (40.08–1129.91)	147.25 (33.96–1766.51)	457.52 (81.42–3842.01)	342.65 (68.06–1787.35)	1213.25 (164.53–13702.16)	0.0043	0.0082
IL10	15.85 (12.64–47.56)	26.52 (12.64–97.40)	21.64 (12.64–98.08)	30.05 (12.64–120.94)	40.66 (12.64–312.22)	0.0933	0.1838
Angiopoietin 2	6219.72 (3124.37–10875.65)	8095.15 (3217.55–17075.40)	6043.98 (3800.03–13005.64)	9916.65 (4746.37–19064.21)	24684.96 (9613.80–38689.72)	<0.0001	<0.0001
TMB	4.23 (3.17–5.55)	4.29 (2.98–6.66)	3.98 (3.39–6.10)	5.31 (3.52–8.33)	6.88 (4.94–10.51)	<0.0001	0.0076
vWF	3303.09 (2360.85–4708.34)	3474.97 (2364.82–5421.20)	3743.15 (2271.03–5215.95)	3851.78 (2599.35–5903.59)	5825.26 (4455.40–8097.86)	<0.0001	0.0002

* *Defined in Table 1*.

### Phenotypes

#### High Risk Phenotypes

**Figure 2:**
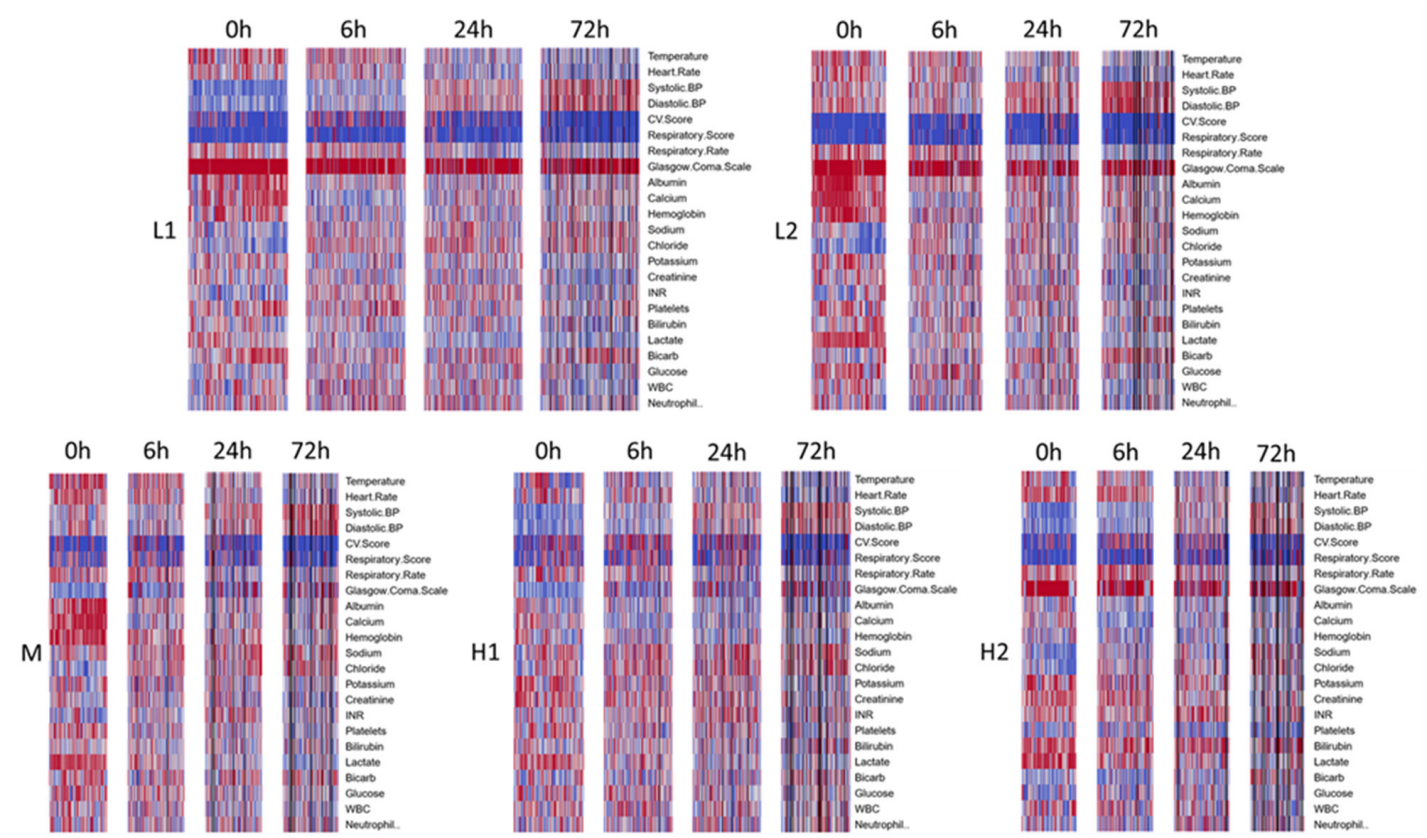
Time evolution of heatmaps, within clusters, for derivation cohort. Types arranged in order of increasing mortality. Profiles for all types get better over time, though notably some abnormalities take longer to normalize or continue to persist in the sicker phenotypes, H1 and H2. Black bars represent patients that died or were discharged prior to that timepoint.

Types H1 and H2 were at the highest risk of death, with 14-day mortality of 28.8% and 36.3% and 60-day mortality of 42.4% and 44.1%, respectively. Type H1, present in 19% of subjects and the oldest group, was the sickest at baseline with the highest APACHE and SOFA scores (Table 4). This group was defined by the presence of multiple organ dysfunction, with especially high degrees of cardiac (81.6%) and respiratory (59.2%) failure. Examination of Figure 1 is notable for low blood pressure, high pressor and MV requirement, low GCS, high creatinine, and high lactate with low serum bicarbonate. Figure 2 demonstrates evolution of the types over time, and it is seen that support increased further at 6 h, particularly with regard to vasopressors as further demonstrated in Supplementary Table S12a. Some improvement is seen at 24 h, and by 72 h, surviving subjects show clear improvement across all variables.

**Table 4:** Demographic and clinical characteristics of phenotypes in derivation cohort.

	**L1** ***N*** **= 178 (28%)**	**L2** ***N*** **= 135 (21%)**	**M** ***N*** **= 102 (16%)**	**H1** ***N*** **= 125 (19%)**	**H2** ***N*** **= 102 (16%)**	***P*** **–value**
Age	58.8 ± 15.6	58.7 ± 15.6	65.6 ± 16.5	65.9 ± 16.5	56.7 ± 14.4	<0.0001
Sex–no. (%)	103 (57.9%)	97 (71.9%)	52 (51.0%)	62 (49.6%)	61 (59.8%)	0.0025
**Race–no. (%)**						
White	121 (68.0%)	81 (60.0%)	73 (71.6%)	81 (64.8%)	68 (66.7%)	0.4077
Black	40 (22.5%)	43 (31.9%)	25 (24.5%)	34 (27.2%)	26 (25.5%)	0.4403
Other	17 (9.6%)	11 (8.1%)	4 (3.9%)	10 (8.0%)	8 (7.8%)	0.5703
**Infectious source– no. (%)**						
Pneumonia	60 (33.7%)	30 (22.2%)	43 (42.2%)	47 (37.6%)	20 (19.6%)	0.0005
Intraabdominal infection	21 (11.8%)	26 (19.3%)	7 (6.9%)	16 (12.8%)	23 (22.5%)	0.0075
Urosepsis	48 (27.0%)	25 (18.5%)	22 (21.6%)	25 (20.0%)	18 (17.6%)	0.2948
Skin or soft tissue infection	7 (3.9%)	15 (11.1%)	6 (5.9%)	9 (7.2%)	11 (10.8%)	0.0987
CNS	2 (1.1%)	1 (0.7%)	2 (2.0%)	1 (0.8%)	0 (0.0%)	0.6862
Endocarditis	0 (0.0%)	0 (0.0%)	0 (0.0%)	0 (0.0%)	0 (0.0%)	N/A
Catheter related infection	7 (3.9%)	4 (3.0%)	1 (1.0%)	4 (3.2%)	5 (4.9%)	0.5853
Unknown	18 (10.1%)	14 (10.4%)	16 (15.7%)	15 (12.0%)	15 (14.7%)	0.5722
Other	11 (6.2%)	14 (10.4%)	3 (2.9%)	6 (4.8%)	10 (9.8%)	0.116
None	4 (2.2%)	6 (4.4%)	2 (2.0%)	2 (1.6%)	0 (0.0%)	0.2193
Blood culture positive– no. (%)	45 (25.3%)	35 (25.9%)	38 (37.3%)	37 (29.6%)	49 (48.0%)	0.0006
**Illness severity**						
APACHEII	17.6 ± 5.4	17.7 ± 6.2	24.1 ± 7.9	28.2 ± 8.1	23.8 ± 6.2	<0.0001
APACHEIII	55.9 ± 16.0	48.5 ± 18.4	71.0 ± 26.3	78.4 ± 24.3	71.2 ± 19.7	<0.0001
Charleson	2.4 ± 2.4	2.2 ± 2.5	2.3 ± 2.2	3.2 ± 2.7	3.2 ± 3.0	0.0021
SOFA	5.8 ± 2.6	5.4 ± 2.8	8.0 ± 3.2	10.2 ± 3.8	9.6 ± 3.1	<0.0001
SOFA cardiac	2.2 ± 1.4	0.9 ± 1.2	1.5 ± 1.6	2.8 ± 1.4	2.2 ± 1.5	<0.0001
SOFA CNS	0.1 ± 0.4	0.2 ± 0.6	2.3 ± 1.2	2.2 ± 1.3	0.4 ± 0.8	<0.0001
SOFA coag	0.5 ± 0.9	0.5 ± 1.0	0.3 ± 0.6	0.5 ± 0.9	1.4 ± 1.2	<0.0001
SOFA liver	0.4 ± 0.8	0.7 ± 1.0	0.4 ± 0.6	0.5 ± 0.8	1.5 ± 1.2	<0.0001
SOFA renal	1.2 ± 1.2	1.5 ± 1.3	1.2 ± 1.2	1.9 ± 1.3	2.2 ± 1.1	<0.0001
SOFA respiratory	1.4 ± 1.0	1.5 ± 1.1	2.4 ± 1.2	2.2 ± 1.2	1.9 ± 1.1	<0.0001
**Physiologic variables**						
SBP	86.9 ± 18.5	114.2 ± 35.2	117.4 ± 34.7	91.7 ± 24.1	90.1 ± 23.9	<0.0001
HR	109.2 ± 22.6	112.6 ± 23.1	121.4 ± 22.8	104.9 ± 26.1	115.3 ± 22.6	<0.0001
Temp	37.6 ± 1.3	37.4 ± 1.4	37.9 ± 1.8	36.9 ± 2.1	37.1 ± 1.5	<0.0001
RR	21.5 ± 5.2	23.2 ± 7.0	24.0 ± 9.1	23.4 ± 8.5	23.0 ± 6.2	0.0289
Tbili	1.1 ± 1.3	1.5 ± 1.9	1.0 ± 0.5	0.9 ± 0.5	3.5 ± 3.4	<0.0001
Lactate	1.9 ± 1.3	3.1 ± 2.1	3.6 ± 3.0	3.3 ± 3.5	6.0 ± 4.7	<0.0001
**Mortality–no. (%)**						
14 days	14 (7.9%)	17 (12.6%)	26 (25.5%)	36 (28.8%)	37 (36.3%)	<0.0001
28 days	17 (9.6%)	19 (14.1%)	33 (32.4%)	43 (34.4%)	42 (41.2%)	<0.0001
60 days	26 (14.6%)	23 (17.0%)	35 (34.3%)	53 (42.4%)	45 (44.1%)	<0.0001
1 year	40 (22.5%)	31 (23.0%)	40 (39.2%)	69 (55.2%)	54 (52.9%)	<0.0001
Multiorgan failure, baseline	66 (37.1%)	55 (40.7%)	75 (73.5%)	100 (80.0%)	88 (86.3%)	<0.0001
**New organ failure–no. (%)**						
Cardiac	125 (70.2%)	46 (34.1%)	48 (47.1%)	102 (81.6%)	80 (78.4%)	<0.0001
Renal	1 (0.6%)	4 (3.0%)	2 (2.0%)	9 (7.2%)	14 (13.7%)	<0.0001
Respiratory	35 (19.7%)	34 (25.2%)	60 (58.8%)	74 (59.2%)	51 (50.0%)	<0.0001
Hosp LOS	10.1 ± 8.9	11.5 ± 10.6	11.1 ± 9.8	13.0 ± 11.5	13.5 ± 14.2	0.076
ICU LOS	3.8 ± 3.2	3.7 ± 4.8	5.6 ± 7.3	5.9 ± 5.8	6.7 ± 8.7	<0.0001
Number of SAEs	4 (2.2%)	3 (2.2%)	4 (3.9%)	2 (1.6%)	2 (2.0%)	0.0502
**Subject Disposition Category–no. (%)**						
Home	114 (64.0%)	87 (64.4%)	42 (41.2%)	34 (27.2%)	46 (45.1%)	<0.0001
SNF	27 (15.2%)	23 (17.0%)	22 (21.6%)	35 (28.0%)	12 (11.8%)	0.013
Dead	16 (9.0%)	17 (12.6%)	30 (29.4%)	43 (34.4%)	40 (39.2%)	<0.0001

Type H2, present in 16% of subjects, comprised the youngest subjects and was unique for an organ failure pattern of liver dysfunction and coagulopathy. These subjects were more likely than other types to have intra-abdominal infection as well as positive blood culture, with nearly half having bacteremia. APACHE scores were high, though nearly identical to type M, the moderate mortality group. Type H2 was also the most likely to develop new renal failure at 13.7% and had a high incidence of new cardiovascular failure at 78.4%. Subjects had the highest rates of preexisting renal failure (26.5%), and chronic liver disease (30.4%). Type H2's low requirement for cardiovascular and respiratory support at baseline is notable. These subjects had the most pronounced lactate elevation, and platelet count and bilirubin were also significantly worse than other groups. Although subjects require pressor support as they progress, respiratory support remains low initially. Despite improvement in some variables, liver dysfunction and coagulopathy persist even at 72 h.

#### Moderate Risk Phenotype

Type M, present in 16% of subjects and one of the oldest groups, was characterized primarily by respiratory failure. This type had 14 and 60-day mortality of 25.5% and 34.3%. Incidence of pneumonia was highest in this type at 42.2%. APACHE scores were high but similar to type H2. At enrollment, respiratory and CNS SOFA scores were elevated similarly to type H1, but the remaining SOFA scores were among the lowest across types. Type M subjects experienced little hypotension at baseline and had a low need of vasopressor support. GCS was low, perhaps from a combination of pre-existing neurological disease and a high need for MV. Respiratory support increases by 6 h along with cardiovascular support to a modest degree but with no significant development of other organ dysfunction. These improve by 24 and 72 h. Serum lactate rapidly normalizes.

#### Low Risk Phenotypes

Type L1, present in 28% of subjects and a younger group, with 14 and 60-day mortality of 7.9% and 14.6%, respectively, represented fluid-refractory shock without multiorgan dysfunction. There is almost universal hypotension in Type L1 subjects and moderate need of vasopressor support, yet little need for respiratory support. Like type M and L2 subjects, albumin, calcium, and hemoglobin are relatively high, and there is no significant dysfunction of other organ systems. Notably, this is the only type without marked lactate elevation. Need for pressor support increased further by 6 h but already decreased significantly by 24 h. These subjects had the lowest incidence of positive blood culture, relatively low illness severity scores, and lowest incidence of new renal and respiratory failure.

Type L2, present in 21% of subjects and comprising a younger group, is best characterized as fluid-responsive shock. Mortality was relatively low in this group, with 12.6% of subjects dying by 14 days and 17% by 60 days. Subjects had a low incidence of bacteremia and some of the lowest illness severity scores. New organ failure was also relatively uncommon. They have few other laboratory abnormalities aside from elevated lactate, which nearly normalizes by 6 h.

**Figure 3:**
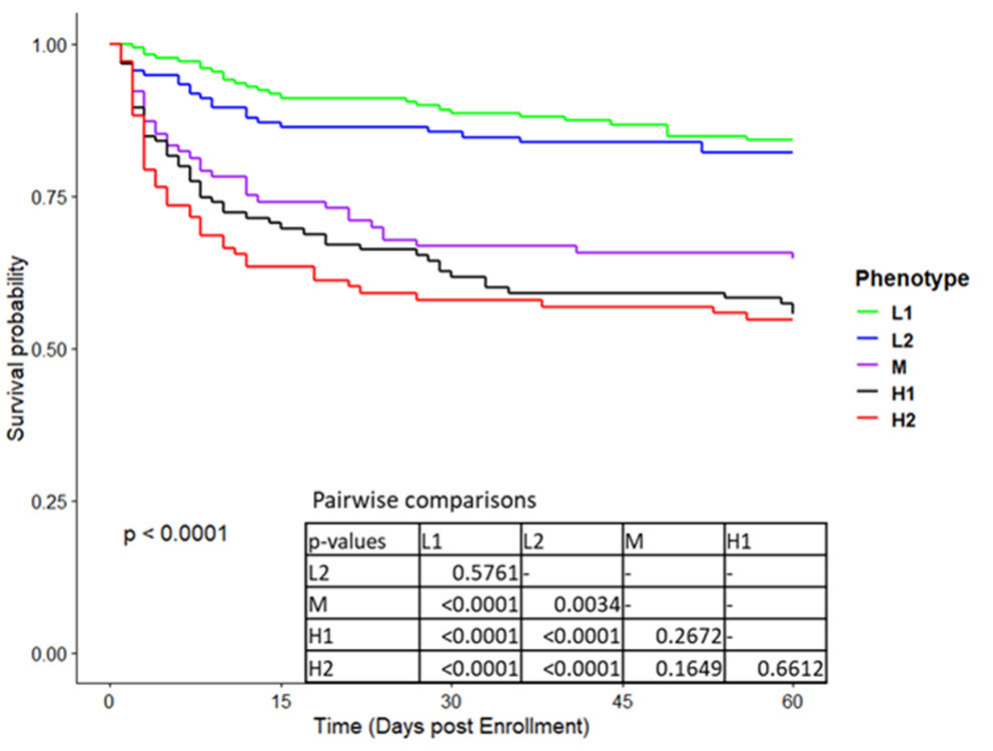
Kaplan Meier survival curves to 60 days for the five phenotypes in derivation cohort.

As can be seen from the Kaplan-Meier curves in Figure 3; Supplementary Figure S4, mortality was significantly different between types at both 60 days and 365 days. For both timepoints, mortality of types H1 and H2 was similar and markedly higher than all other types.

### Validation Cohort

The analysis performed on the validation cohort yielded similar patterns of organ failure and mortality (Figure 1B; Supplementary Figure S5; Supplementary Tables S2–S4; Supplementary Table S12b) compared to the derivation cohort, with the exception that group L2 (fluid-responsive shock) had the lowest 60-day mortality (13.4%). Group L1 (fluid-refractory shock without multiorgan dysfunction) could also be identified in validation, with hypotension, pressor requirement, lowest lactates, and lack of consistent organ dysfunction; 60-day mortality was 18.3%. The M group (respiratory failure group without significant involvement of other systems) was once again the moderate mortality group, with 60-day mortality of 24%. This group was notably smaller in the validation cohort. Once again, two high mortality groups were observed with the same patterns of organ failure observed in the derivation analysis, H1 (multiple organ dysfunction including respiratory failure, cardiovascular collapse, CNS depression, and AKI), and H2 (hepatobiliary dysfunction and coagulopathy). Mortality for these two groups at 60 days was 37% and 46%, respectively.

### Cytokines and Other Plasma Markers

Following definition of the five phenotypes based on clinical markers alone, we examined available measurements of the cytokines IL6, IL10, and TNF as well as the plasma markers angiopoietin-2, thrombomodulin (TMB), and vWF within the confines of each phenotype (Table 1). Although they were not measured with enough frequency to permit inclusion in the clustering analysis, they provided the basis for exploratory comparisons. Type H2 has the highest measurements for all markers, though differences did not reach significance for TNF and IL10. Direct comparison of types H1 and H2 revealed that H2 remained significantly higher for IL6, angiopoietin-2, TMB, and vWF. Angiopoietin-2 and TMB have been associated with endothelial dysfunction and capillary leak (17–19). VWF is a marker of endothelial injury and has been demonstrated to be elevated in subjects with disseminated intravascular coagulation (20). Thus, type H2 appeared to be more inflamed and have a greater degree of activation of both the endothelial and coagulation systems than any other type, including type H1.

### Multinomial Model

As described above, a multinomial model was developed to be able to identify types at baseline using a more parsimonious number of variables. Twelve variables were ultimately selected based on the results of the general linearized models: Lactate, Temperature, Heart Rate, Systolic BP, Diastolic BP, GCS, Sodium, Chloride, Bicarbonate, Calcium, Albumin, Hemoglobin. An advantage of this set is that the only labs needed would likely be obtained on all patients with sepsis early and does not require liver function tests or coagulation studies. Model coefficients for type prediction, with M as the reference type, are provided in Supplementary Table S9. Overall accuracy was 83.8% in the derivation cohort and 65.6% in the validation cohort. Complete performance in the validation cohort is shown in Table 5, with results in the derivation cohort provided in Supplementary Table S10. In the validation cohort, for subjects predicted to be low risk by the model (types L1 or L2), fewer than 20% were actually high risk. The model had greatest difficulty differentiating types M and H1, likely due to the high prevalence of respiratory failure and neurologic depression in both groups, as well as some possibility of misclassification by the clustering analysis given the smaller number of subjects in the validation cohort.

**Table 5:** Multinomial model accuracy in the validation cohort.

**Predicted**	**Actual phenotype**					**Predicted total**	**High risk** **mis–predicted**
	**L1**	**L2**	**M**	**H1**	**H2**		
l1	87 (80%)	35 (36%)	0 (0%)	1 (1%)	10 (2%)	133	0.082707
l2	9 (8%)	56 (58%)	0 (0%)	1 (1%)	12 (24%)	78	0.166667
m	0 (0%)	2 (2%)	24 (96%)	26 (26%)	1 (2%)	53	0.509434
h1	3 (3%)	2 (2%)	0 (0%)	57 (57%)	1 (2%)	63	
h2	10 (9%)	2 (2%)	1 (4%)	15 (15%)	26 (52%)	54	
Actual total	109	97	25	100	50		

### Impact of Fluid Administration

**Figure 4:**
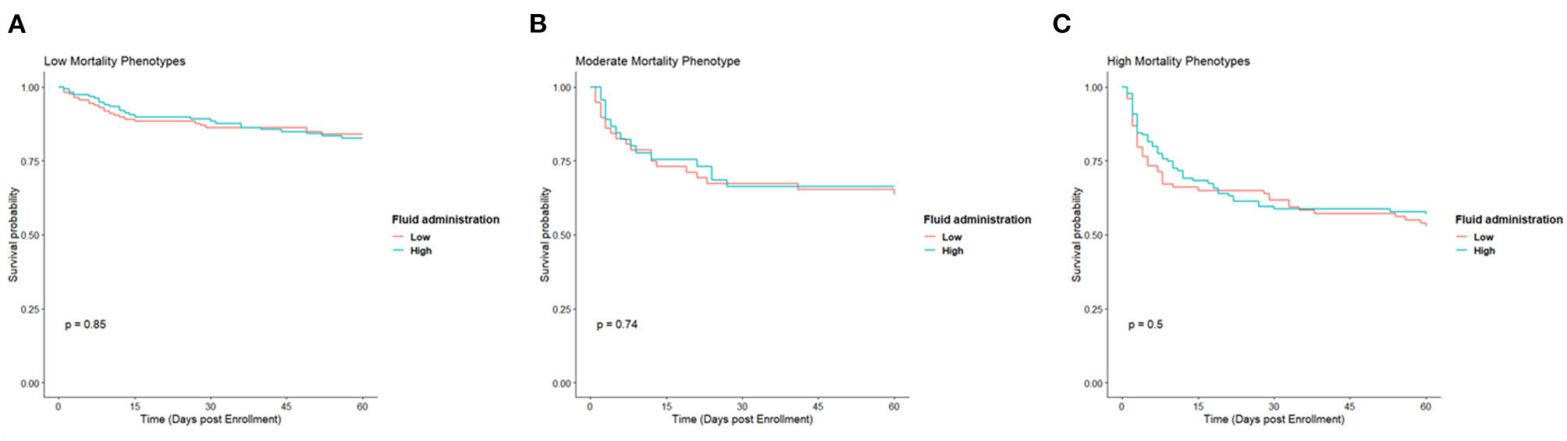
Kaplan Meier survival curves for low **(A)**, moderate **(B)**, and high **(C)** mortality phenotypes based on whether they received a high amount of fluid or low amount in the first 24 h.

We sought to evaluate whether a treatment effect could be observed based on type. Since no treatment effect was seen between treatment arms, we dichotomized based on the amount of fluid administered in the first 24 h. The median amount of fluid received was approximately six liters (Supplementary Figure S6), so the two groups were defined as follows: “high fluid” were subjects that received >6 L, and “low fluid” were subjects that received <6 L. The high and low mortality types were each combined together for comparison to each other and the M type. There was no difference between high and low fluid administration in any of the types (Figure 4).

## Discussion

It has become increasingly clear in sepsis research and clinical experience that there is much phenotypic variability in patients with sepsis that likely needs to be accounted for when testing sepsis-targeted therapies. Indeed, the presumption is that phenotypes relate to mechanism of disease driven by underlying endotypes, and thus are an early indicator of actionability, although further study is required to explore this idea. The goal of this retrospective analysis of a large multicenter study of subjects with known septic shock was to explore whether subgroups of patients could be identified based solely on clinical data using unsupervised learning techniques. We found five distinct clinical phenotypes consisting of fluid-responsive septic shock (type L2), fluid-refractory septic shock without multiorgan failure (type L1), septic shock with respiratory failure (type M), septic shock with multiorgan dysfunction (type H1), and septic shock with hepatobiliary dysfunction and coagulopathy (type H2). Most notably, we identified two types, H1 and H2, with similarly high mortality but distinct baseline illness severity scores, clinical characteristics including age, organ failure patterns, and plasma marker elevations. These two types also progressed in time differently, with type H2 continuing to show higher bilirubin, lower platelet count, higher INR, and even a trend toward more persistent shock at 72 h while a significant proportion of type H1 subjects demonstrated recovery by that time. This identification of two high risk types with differing trajectories is particularly noteworthy given that many previous efforts at phenotyping sepsis have often resulted in identification of one high mortality type with significant cytokine elevation and no separation of organ failure patterns beyond single and multiple organ failure. However, by analyzing specifically subjects with septic shock rather than all subjects with sepsis, we provide increased detail in distinguishing clinical features across types.

Our analysis is most directly comparable to other clinical phenotyping studies. Some of the study populations in these analyses have included all sepsis patients while others have consisted of only septic shock. Additionally, some studies have derived their subjects from EMR-based cohorts, while others utilized trial populations. There are advantages and disadvantages of both means of deriving study populations. EMR-based cohorts typically offer larger study populations but may have less granular data as well as decreased certainty in the diagnosis, whereas trial populations commonly have fewer subjects but certainty of sepsis or septic shock diagnosis and often highly regimented data (21).

We believe this study is complementary to a recently published EHR-based study by Seymour, et al. defining four broad phenotypes of sepsis defined by a SEPSIS-3 based computable phenotype across all hospitalized patients (12). This study defined four broad phenotypes of sepsis based on all hospitalized patients. They then leveraged this analysis to apply these phenotypes to several other cohorts, including the ProCESS study population. However, because the types were derived based on all sepsis patients, rather than those with septic shock, there was less differentiation among the highest risk patients, in whom there is the greatest opportunity to impact mortality. Indeed, the highest mortality group, delta, combines the organ dysfunctions from both of the highest mortality groups of this study (Supplementary Table S11). Others have also attempted to identify clinical phenotypes of sepsis in both study populations and EMR-derived cohorts. Knox, et al. specifically examined severe sepsis and septic shock in a retrospectively identified EMR-derived cohort using self-organizing maps to identify clinical phenotypes (22). The baseline measurements relied on a fairly wide time window of 6 hours before to 24 h after ICU admission. The analysis produced four clusters which they describe as “(1) shock with elevated creatinine, (2) minimal MODS, (3) shock with hypoxemia and altered mental status, and (4) hepatic disease”. Mortality was highest in cluster 3 followed by cluster 4. Of note, cluster 4, that characterized by hepatic disease, also was most associated with coagulation dysfunction similar to type H2 from this study, while cluster 3 had a high degree of concomitant cardiovascular dysfunction similar to type H1. Our analysis further elucidates these types however by also providing information about how they progress in time, as well as demonstrating that they can be identified even earlier in the course of illness, when interventions may have the greatest impact. Zhang, et al. utilized the MIMIC-III database in combination with latent profile analysis to identify 4 clinical phenotypes in sepsis, similarly to SENECA, also with a single high mortality group demonstrating multiple organ dysfunction and comprising 11% of the cohort (23). The other three groups either showed no major overarching dysfunction or single organ dysfunction. Their results also demonstrate a difficulty in differentiating the most severely ill patients when all sepsis patients are considered together. Perhaps most similarly to our study population, Gårdlund, et al., used latent class analysis in septic shock subjects from the PROWESS study and identified 6 clinical phenotypes, with some similarity to our 5 types (24). Their Class 2, Pneumonia with ARDS, bears significant similarity to type M from our analysis, with a high degree of respiratory failure but little other organ dysfunction. Class 4, Severe septic shock, has multiple similarities to type H2 with fairly minimal cardiovascular dysfunction but abnormalities of the coagulation, renal, and hepatic systems, as well as thrombocytopenia. Class 5, Pneumonia with ARDS and MODS, has multiple similarities to our type H1, with marked respiratory and renal dysfunction as well as some cardiovascular dysfunction and much less coagulation abnormality than class 4. We did not identify a type similar to the highest mortality group, Class 6: Late septic shock, but notably the majority of these subjects were already admitted to the hospital and thus may represent a group absent from the ProCESS study population, which enrolled subjects presenting to the emergency department.

Thus, our contribution drills down to a subgroup of subjects with confirmed septic shock and provides further evidence of unique moderate and high mortality groups that likely have distinct physiological derangements. Furthermore, unique to our analysis, we show how these types evolve in time and yet maintain the same signature of organ dysfunctions. Similar to how we focused on what is essentially a subset of sepsis subjects, it is likely that further study of the sickest phenotypes could yield further subgroups. This is suggested from work by Carcillo, et al., who recently identified three inflammation phenotypes in children with severe sepsis, with those patients showing increased incidence of macrophage activation syndrome (25). Lastly, this work should be considered parallel to endotype work by other groups such as Wong, et al. (26–28). Transcriptomic work such as theirs aims to describe sepsis phenotypes in mechanistic terms, and it is expected that the endotypes they identify would map to clinical phenotypes. Hierarchical agglomerative clustering was chosen as the means of cluster identification. Typically, there remains a degree of arbitrariness in choosing optimal cluster number, a concern also present for other unsupervised clustering techniques. We mitigated this issue by using consensus clustering to both identify a number of clusters data can support and validate cluster membership. We believe this approach results in more robust clusters, and the observation of five distinct phenotypes identified purely with unsupervised learning techniques lends credibility to their robustness. Furthermore, these clusters were demonstrated in a smaller validation cohort and matched well to the originally defined types. This is in contrast to EHR-derived phenotypes that sometimes lead to less-than-ideal matches in their validation cohorts. Indeed, a recent analysis demonstrated that application of various sepsis definitions to EHR data can lead to markedly different cohorts (29). By relying on a cohort of trial-vetted sepsis patients, we were likely able to mitigate this uncertainty. Further, clustering was performed on ranked values, which permitted increased discrimination between measurements while limiting the impact of outliers. However, it must be noted that were this analysis to be applied to a new population of unknown severity, it would need to be mapped to a standard scale.

Following the identification of clinical phenotypes, we examined plasma marker profiles across types and found significant differences. IL6 levels in particular were highest in type H2 at time of presentation, and markers of endothelial dysfunction and coagulation were significantly higher in this type as well. This further supports that types H1 and H2 represent distinct groups with differing underlying pathophysiological phenomena despite their similarly high mortality.

Most important for an analysis such as this is clinical applicability. The most effective use of this sort of exercise would likely be an EHR-based tool that calculates likelihood of type membership, perhaps by distance to the centroid of each cluster. Given that types were identified entirely by baseline characteristics, this would allow randomization of patients with septic shock early in their course to allow comparison of targeted therapies, rather than the one-size-fits-all approach that has been employed, mostly unsuccessfully, thus far in sepsis trials. Importantly, such an approach might allow reduction of potential harm of immunomodulating therapies. Presumably, type L1 represent subjects with a very robust, but appropriate response to infection, with rapid resolution of shock and vasopressor requirement. Taming the immune response might harm this type but may be useful in type H2, which demonstrated the highest levels of IL6. Similarly, therapies aimed at reducing angiopoietin-2 activity are actively being investigated in pre-clinical models and have shown early promise (30). With the highest levels of angiopoietin-2 of any type, H2 appears to be most likely to benefit from these therapies. Where an EHR-based tool is not feasible, one could implement a multinomial model that utilizes a more limited set of data, as discussed above. Thus, the actionability of clinical phenotyping as presented lies in the enhanced ability to target therapies, or predictive enrichment of clinical trials, that is, the focusing of therapies to phenotypes with profiles suggesting an enhanced probability of positive response (31, 32). As of yet, no efforts have been made to apply phenotypes such as ours to sepsis trials, though this is clearly the next step forward. Part of this is due to lack of wide validation of individual typing studies, as well as a reliable means of identifying types early in the course. Our results show some agreement with those of other septic shock phenotyping studies, bolstering the evidence base for their existence. Moreover, we provide evidence that types could be identified with data available early in the course, and that these types are unlikely to change over the course of treatment.

As with any retrospective study, there are weaknesses to our results. There was a degree of missingness to the data that required imputation to be employed. For most markers, the amount of missing data was quite small with 0 to 2% of data imputed for 14 of the included markers. However, GCS, neutrophil percentage, and bicarb had approximately 10% missingness, and INR, in particular, was missing for about 25% of patients at baseline. However, it was strongly felt that a marker of coagulation needed to be included. In comparison to other studies of this nature, our missingness criteria were either similar or more stringent. Additionally, conclusions about cytokine and other plasma marker trends are limited by sparser measurements, with data present for 43% to 49% of patients within a given type. This very high degree of missingness precluded their inclusion in the clustering analysis and limits interpretation of these trends, as well as the confidence in potential treatment targets such as angiopoietin-2. Next, the multinomial model fared well in the derivation cohort, with accuracy of approximately 84%, but was much lower at 66% in the validation cohort. Some of this reduced accuracy may have been due to the potential for misclassification by the clustering analysis in the validation cohort due to its smaller sample size. At a minimum, it serves as a proof of concept, but would likely need further evaluation before being used as a prospective classification tool. Lastly, there is some limitation to generalizability of the analysis given that the validation cohort was derived from the same study population, albeit from geographically distinct sites, as the derivation cohort. The enrollment criteria ensured that all included subjects were part of a curated septic shock cohort, but it does mean that all subjects met these same criteria. Early management would also have had similarities given that it would have been partly dictated by the study protocol. However, subsequent management would have likely been guided by institutional practice, and since hospitals themselves were randomized, rather than subjects irrespective of site, these variations in practice would have remained independent of each other, resulting in cohorts that were effectively separate for most of the management of these subjects.

## Conclusions

This study extends prior results of phenotyping of inpatient sepsis, focusing on subjects with clinically proven septic shock at baseline. We identified five distinct clinical phenotypes with distinct presentation, and perhaps most notably, evolution in time. Such phenotyping presents an opportunity for early clinical actionability. Further studies exploring the correlation between these phenotypes and mechanism-based sepsis endotyping are necessary.

## Data Availability Statement

The data analyzed in this study is subject to the following licenses/restrictions. Portions of the dataset may be shareable under Data Use agreements with the University of Pittsburgh. Requests to access these datasets should be directed to cler@pitt.edu.

## Ethics Statement

Ethical review and approval was not required for the study on human participants in accordance with the local legislation and institutional requirements. Written informed consent for participation was not required for this study in accordance with the national legislation and the institutional requirements.

## Author Contributions

GC: data acquisition. ZA, LZ, RP, HG, IB, DS, and GC: conceptual foundation. ZA, AU, LZ, and GC: analyses. ZA, LZ, DS, RP, IB, and GC: interpretation. ZA, LZ, and GC: manuscript write-up. ZA, LZ, DS, RP, HG, IB, and GC: manuscript review. All authors contributed to the article and approved the submitted version.

## Funding

This work was supported by the National Institutes of Health [grant number RO1 GM105728]. The sponsor had no role in the study design; collection, analysis, and interpretation of data; writing of the report; and decision to submit the article for publication.

## Conflict of Interest

The authors declare that the research was conducted in the absence of any commercial or financial relationships that could be construed as a potential conflict of interest.

## Publisher's Note

All claims expressed in this article are solely those of the authors and do not necessarily represent those of their affiliated organizations, or those of the publisher, the editors and the reviewers. Any product that may be evaluated in this article, or claim that may be made by its manufacturer, is not guaranteed or endorsed by the publisher.

## Abbreviations

BiPAP: Bilevel positive airway pressure
BP: blood pressure
CPAP: continuous positive airway pressure
EHR: electronic health record
GCS: Glasgow Coma Scale
IL6: interleukin 6
IL10: interleukin 10
MICE: Multivariate Imputation by Chained Equations
MV: mechanical ventilation
PEEP: positive end expiratory pressure
ProCESS: Protocolized Care for Early Septic Shock
SOFA: sequential organ failure assessment
TMB: thrombomodulin
TNF: tumor necrosis factor
vWF: von Willebrand factor.
