# Supplementary material for: Identification of Clinical Phenotypes in Septic Patients Presenting With Hypotension or Elevated Lactate
Source: Front Med (Lausanne). 2022 May 19;9:794423. doi: 10.3389/fmed.2022.794423 (PMC9160971; doi:10.3389/fmed.2022.794423)
Supplement: Supplementary file 1 [file Data_Sheet_1.docx]

Supplementary Material

# Supplementary Tables

**Table S1:** Pre-existing conditions and medications taken in the prior week in derivation cohort. Phenotypes H1 and H2 were specifically compared against each other using Tukey tests because these were the two highest mortality groups

|  | **L1**  **N=178 (28%)** | **L2**  **N=135 (21%)** | **M**  **N=102 (16%)** | **H1**  **N=125 (19%)** | **H2**  **N=102 (16%)** | **All** | ***P*-value** | **H1 vs H2*** |
| --- | --- | --- | --- | --- | --- | --- | --- | --- |
| **Chronic Conditions (% having)** |  |  |  |  |  |  |  |  |
| **Hypertension** | 59.6 | 59.3 | 61.8 | 64.8 | 42.2 | 58.1 | 0.0079 | 0.0051 |
| **Diabetes Mellitus** | 30.3 | 38.5 | 40.2 | 40.8 | 28.4 | 35.4 | 0.1183 | 0.2959 |
| **Dementia** | 2.2 | 3 | 20.6 | 20.8 | 2 | 8.9 | 0 | 0 |
| **Myocardial Infarction** | 11.8 | 7.4 | 5.9 | 8.8 | 6.9 | 8.6 | 0.4174 | 0.9855 |
| **Congestive Heart Failure** | 12.9 | 8.9 | 10.8 | 14.4 | 11.8 | 11.8 | 0.6971 | 0.9735 |
| **Cerebral Vascular Disease** | 7.9 | 5.9 | 19.6 | 15.2 | 2 | 9.8 | 0 | 0.0066 |
| **Plegia** | 6.2 | 5.2 | 10.8 | 8 | 1 | 6.2 | 0.0526 | 0.187 |
| **Peripheral Vascular Disease** | 7.9 | 4.4 | 8.8 | 9.6 | 7.8 | 7.6 | 0.5838 | 0.9878 |
| **Respiratory Disease** | 28.1 | 14.8 | 19.6 | 25.6 | 17.6 | 21.8 | 0.0343 | 0.5958 |
| **Pleural Effusion** | 7.7 | 3.5 | 12.8 | 13.6 | 17.4 | 10.3 | 0.0085 | 0.905 |
| **Cancer** | 14.6 | 15.6 | 6.9 | 19.2 | 17.6 | 15 | 0.1049 | 0.9975 |
| **AIDS** | 3.4 | 2.2 | 2 | 3.2 | 6.9 | 3.4 | 0.2975 | 0.5571 |
| **Renal failure** | 17.4 | 12.6 | 9.8 | 20.8 | 26.5 | 17.3 | 0.0102 | 0.79 |
| **Dialysis** | 5.6 | 5.2 | 6.9 | 6.4 | 10.8 | 6.7 | 0.4685 | 0.6833 |
| **Cirrhosis** | 3.9 | 3.7 | 1 | 4 | 25.5 | 6.9 | 0 | 0 |
| **Chronic Liver Disease** | 4.5 | 5.9 | 2.9 | 5.6 | 30.4 | 8.9 | 0 | 0 |
| **Connective Tissue Disease** | 5.1 | 0 | 3.9 | 4 | 3.9 | 3.4 | 0.1657 | 1 |
| **Medications within preceding week (% receiving)** |  |  |  |  |  |  |  |  |
| **Antibiotics** | 26.4 | 17 | 12.7 | 27.2 | 28.4 | 22.7 | 0.0112 | 0.9995 |
| **Aspirin** | 25.8 | 23 | 30.4 | 25.6 | 19.6 | 24.9 | 0.4708 | 0.8381 |
| **Steroids** | 21.9 | 11.1 | 12.7 | 17.6 | 13.7 | 16 | 0.0766 | 0.9325 |
| **Anticoagulants** | 15.2 | 11.9 | 19.6 | 24.8 | 10.8 | 16.4 | 0.0186 | 0.0358 |
| **Statins** | 27.5 | 18.5 | 35.3 | 26.4 | 17.6 | 25.1 | 0.0141 | 0.5483 |
| **Beta blockers** | 30.3 | 28.9 | 32.4 | 32.8 | 22.5 | 29.6 | 0.48 | 0.4464 |

**Table S2:** Demographic and clinical characteristics of phenotypes in validation cohort

|  | **L1**  **N=109 (29%)** | **L2**  **N=97 (25%)** | **M**  **N=25 (7%)** | **H1**  **N=100 (26%)** | **H2**  **N=50 (13%)** | ***P*-value** |
| --- | --- | --- | --- | --- | --- | --- |
| **Age** | 61.2±17.1 | 62.3±15.7 | 62.3±19.1 | 65.6±14.6 | 54.5±12.2 | 0.0021 |
| **Sex - no. (%)** | 52 (47.7%) | 55 (56.7%) | 16 (64.0%) | 52 (52.0%) | 33 (66.0%) | 0.1956 |
| **Race - no. (%)** |  |  |  |  |  |  |
| **White** | 82 (75.2%) | 74 (76.3%) | 16 (64.0%) | 77 (77.0%) | 32 (64.0%) | 0.3223 |
| **Black** | 16 (14.7%) | 18 (18.6%) | 7 (28.0%) | 21 (21.0%) | 15 (30.0%) | 0.1858 |
| **Other** | 11 (10.1%) | 5 (5.2%) | 2 (8.0%) | 2 (2.0%) | 3 (6.0%) | 0.1755 |
| **Infectious source - no. (%)** |  |  |  |  |  |  |
| **Pneumonia** | 26 (23.9%) | 40 (41.2%) | 10 (40.0%) | 33 (33.0%) | 14 (28.0%) | 0.0837 |
| **Intraabdominal Infection** | 14 (12.8%) | 8 (8.2%) | 3 (12.0%) | 16 (16.0%) | 12 (24.0%) | 0.1166 |
| **Urosepsis** | 32 (29.4%) | 18 (18.6%) | 3 (12.0%) | 20 (20.0%) | 8 (16.0%) | 0.1415 |
| **Skin or soft.tissue infection** | 8 (7.3%) | 9 (9.3%) | 0 (0.0%) | 8 (8.0%) | 2 (4.0%) | 0.4817 |
| **CNS** | 0 (0.0%) | 0 (0.0%) | 1 (4.0%) | 1 (1.0%) | 0 (0.0%) | 0.1089 |
| **Endocarditis** | 5 (4.6%) | 0 (0.0%) | 0 (0.0%) | 0 (0.0%) | 0 (0.0%) | 0.0132 |
| **Catheter related infection** | 5 (4.6%) | 2 (2.1%) | 1 (4.0%) | 1 (1.0%) | 0 (0.0%) | 0.3215 |
| **Unknown** | 11 (10.1%) | 9 (9.3%) | 4 (16.0%) | 15 (15.0%) | 6 (12.0%) | 0.6797 |
| **Other** | 6 (5.5%) | 6 (6.2%) | 0 (0.0%) | 5 (5.0%) | 7 (14.0%) | 0.1302 |
| **None** | 2 (1.8%) | 5 (5.2%) | 3 (12.0%) | 1 (1.0%) | 1 (2.0%) | 0.0396 |
| **Blood Culture Positive - no. (%)** | 36 (33.0%) | 19 (19.6%) | 6 (24.0%) | 32 (32.0%) | 22 (44.0%) | 0.0298 |
| **Illness Severity** |  |  |  |  |  |  |
| **APACHEII** | 19.2±5.9 | 15.9±5.2 | 19.6±7.1 | 25.5±8.5 | 21.2±5.7 | 0 |
| **APACHEIII** | 59.9±14.1 | 51.6±15.1 | 63.5±19.9 | 76.3±24.5 | 67.5±19.7 | 0 |
| **Charleson** | 2.6±2.6 | 2.3±2.2 | 1.9±2.0 | 3.0±2.7 | 3.3±3.3 | 0.0851 |
| **SOFA** | 6.8±2.8 | 4.4±2.4 | 6.9±3.0 | 9.9±3.4 | 8.9±3.2 | 0 |
| **SOFA Cardiac** | 3.0±1.4 | 1.4±1.5 | 1.3±1.7 | 3.0±1.5 | 2.6±1.6 | 0 |
| **SOFA CNS** | 0.1±0.3 | 0.2±0.5 | 1.7±1.0 | 1.9±1.3 | 0.1±0.3 | 0 |
| **SOFA Coag** | 0.5±0.9 | 0.2±0.6 | 0.3±0.9 | 0.6±1.0 | 1.0±1.4 | 0 |
| **SOFA Liver** | 0.4±0.7 | 0.3±0.6 | 0.4±0.8 | 0.7±1.0 | 1.2±1.2 | 0 |
| **SOFA Renal** | 1.5±1.3 | 0.8±1.0 | 0.8±1.0 | 1.4±1.2 | 2.0±1.2 | 0 |
| **SOFA Respiratory** | 1.3±1.0 | 1.5±1.0 | 2.3±1.2 | 2.4±1.2 | 1.9±1.3 | 0 |
| **Physiologic variables** | | |  |  |  |  |
| **SBP** | 90.1±20.3 | 107.7±23.8 | 138.0±37.0 | 99.6±23.7 | 98.4±21.9 | 0 |
| **HR** | 105.5±23.6 | 112.3±20.5 | 128.8±21.8 | 105.0±22.8 | 112.9±27.3 | 0 |
| **Temp** | 37.5±1.4 | 37.2±1.3 | 37.9±1.6 | 37.2±1.6 | 37.0±1.1 | 0.0429 |
| **RR** | 21.1±5.6 | 21.9±5.0 | 23.3±6.5 | 22.7±7.7 | 22.0±4.9 | 0.3254 |
| **Tbili** | 1.2±1.1 | 0.9±0.6 | 1.2±1.0 | 1.3±1.8 | 3.8±5.8 | 1.00E-04 |
| **Lactate** | 1.8±1.2 | 1.8±1.1 | 1.8±1.1 | 2.8±2.2 | 4.6±2.7 | 0 |
| **Mortality - no. (%)** | |  |  |  |  |  |
| **14 days** | 12 (11.0%) | 4 (4.1%) | 3 (12.0%) | 25 (25.0%) | 17 (34.0%) | 0 |
| **28 days** | 15 (13.8%) | 11 (11.3%) | 3 (12.0%) | 33 (33.0%) | 18 (36.0%) | 0 |
| **60 days** | 20 (18.3%) | 13 (13.4%) | 6 (24.0%) | 37 (37.0%) | 23 (46.0%) | 0 |
| **1 year** | 32 (29.4%) | 20 (20.6%) | 8 (32.0%) | 50 (50.0%) | 25 (50.0%) | 0 |
| **Multiorgan Failure, Baseline** | 56 (51.4%) | 21 (21.6%) | 17 (68.0%) | 82 (82.0%) | 40 (80.0%) | 0 |
| **New Organ Failure - no. (%)** |  |  |  |  |  |  |
| **Cardiac** | 87 (79.8%) | 29 (29.9%) | 11 (44.0%) | 83 (83.0%) | 37 (74.0%) | 0 |
| **Renal** | 1 (0.9%) | 1 (1.0%) | 0 (0.0%) | 1 (1.0%) | 6 (12.0%) | 1.00E-04 |
| **Respiratory** | 19 (17.4%) | 13 (13.4%) | 12 (48.0%) | 55 (55.0%) | 23 (46.0%) | 0 |
| **Hosp LOS** | 10.5±8.1 | 9.2±7.5 | 10.8±10.6 | 14.1±14.0 | 10.9±10.4 | 0.0185 |
| **ICU LOS** | 4.4±4.0 | 3.1±3.9 | 4.9±6.1 | 7.9±11.0 | 5.2±5.8 | 0 |
| **Number of SAEs** | 3 (2.8%) | 0 (0.0%) | 0 (0.0%) | 6 (6.0%) | 1 (2.0%) | 0.1882 |
| **Subj Disposition Category - no. (%)** | |  |  |  |  |  |
| **Home** | 72 (66.1%) | 62 (63.9%) | 12 (48.0%) | 34 (34.0%) | 20 (40.0%) | 0 |
| **SNF** | 14 (12.8%) | 17 (17.5%) | 5 (20.0%) | 21 (21.0%) | 5 (10.0%) | 0.3556 |
| **Dead** | 15 (13.8%) | 7 (7.2%) | 4 (16.0%) | 32 (32.0%) | 19 (38.0%) | 0 |

**Table S3:** Pre-existing conditions and medications taken in the prior week in validation cohort. Phenotypes H1 and H2 were specifically compared against each other using Tukey tests because these were the two highest mortality groups

|  | **L1**  **N=109** | **L2**  **N=97** | **M**  **N=25** | **H1**  **N=100** | **H2**  **N=50** | **All** | ***P*-value** | **H1 vs H2*** |
| --- | --- | --- | --- | --- | --- | --- | --- | --- |
| **Chronic Conditions (% having)** |  |  |  |  |  |  |  |  |
| **Hypertension** | 62.4 | 59.8 | 48 | 63 | 48 | 59.1 | 0.2939 | 1 |
| **Diabetes Mellitus** | 26.6 | 43.3 | 28 | 35 | 34 | 34.1 | 0.146 | 0.9855 |
| **Dementia** | 1.8 | 7.2 | 20 | 12 | 0 | 6.8 | 0.0007 | 0.0093 |
| **Myocardial Infarction** | 14.7 | 9.3 | 16 | 9 | 14 | 11.8 | 0.5835 | 0.9991 |
| **Congestive Heart Failure** | 14.7 | 13.4 | 8 | 12 | 6 | 12.1 | 0.5633 | 0.9991 |
| **Cerebral Vascular Disease** | 1.8 | 16.5 | 8 | 14 | 0 | 8.9 | 0.0002 | 0.7675 |
| **Plegia** | 4.6 | 0 | 0 | 6 | 2 | 3.1 | 0.1081 | 0.99 |
| **Peripheral Vascular Disease** | 5.5 | 9.3 | 8 | 14 | 6 | 8.9 | 0.2591 | 0.9985 |
| **Respiratory Disease** | 24.8 | 21.6 | 0 | 21 | 8 | 19.2 | 0.012 | 0.9184 |
| **Pleural Effusion** | 16.3 | 11.6 | 4.5 | 15.8 | 16.7 | 14.3 | 0.5561 | 0.6653 |
| **Cancer** | 28.4 | 15.5 | 20 | 21 | 30 | 22.8 | 0.1535 | 0.8665 |
| **AIDS** | 2.8 | 1 | 0 | 0 | 6 | 1.8 | 0.0916 | 0.3571 |
| **Renal failure** | 21.1 | 7.2 | 4 | 15 | 24 | 15.2 | 0.0101 | 0.1478 |
| **Dialysis** | 5.5 | 1 | 4 | 6 | 6 | 4.5 | 0.4365 | 0.9949 |
| **Cirrhosis** | 6.4 | 1 | 0 | 11 | 12 | 6.6 | 0.0153 | 0.269 |
| **Chronic Liver Disease** | 5.5 | 5.2 | 0 | 10 | 10 | 6.8 | 0.2962 | 0.4862 |
| **Connective Tissue Disease** | 8.3 | 6.2 | 0 | 2 | 2 | 4.7 | 0.1263 | 0.9953 |
| **Medications within preceding week (% receiving)** |  |  |  |  |  |  |  |  |
| **Antibiotics** | 26.6 | 26.8 | 8 | 23 | 26 | 24.4 | 0.3558 | 0.4294 |
| **Aspirin** | 33.9 | 29.9 | 32 | 40 | 22 | 32.8 | 0.2467 | 0.9077 |
| **Steroids** | 25.7 | 25.8 | 4 | 18 | 16 | 21 | 0.0769 | 0.7466 |
| **Anticoagulants** | 25.7 | 17.5 | 12 | 26 | 12 | 21 | 0.1203 | 1 |
| **Statins** | 38.5 | 35.1 | 16 | 32 | 24 | 32.5 | 0.1445 | 0.9567 |
| **Beta blockers** | 42.2 | 28.9 | 20 | 35 | 22 | 32.8 | 0.044 | 0.9998 |

**Table S4:** Comparison of clustered clinical variables and molecular markers across phenotypes at baseline in the validation cohort. Phenotypes H1 and H2 were specifically compared against each other using Tukey tests because these were the two highest mortality groups

|  | L1  N=109 (29%) | L2  N=97 (25%) | M  N=25 (7%) | H1  N=100 (26%) | H2  N=50 (13%) | *P*-value | H1 vs H2 |
| --- | --- | --- | --- | --- | --- | --- | --- |
| Clustered clinical variables, mean±SD | | | | | | | |
| Temperature | 37.31 (1.09) | 37.33 (1.02) | 38.26 (1.40) | 37.13 (1.18) | 36.94 (1.08) | 0.0001 | <0.0001 |
| Heart rate | 102.29 (18.48) | 106.45 (19.74) | 119.72 (17.38) | 102.96 (21.48) | 113.72 (17.63) | <0.0001 | 0.7169 |
| Systolic BP | 82.25 (12.39) | 104.18 (18.66) | 115.72 (21.17) | 93.24 (19.01) | 90.44 (13.89) | <0.0001 | <0.0001 |
| Diastolic BP | 45.52 (7.93) | 58.26 (11.05) | 65.60 (13.87) | 49.56 (11.05) | 54.20 (10.48) | <0.0001 | 0.0001 |
| Cardiovascular score | 1.66 (1.61) | 0.35 (0.98) | 0.84 (1.43) | 1.63 (1.60) | 1.18 (1.59) | <0.0001 | 0.8755 |
| Respiratory score | 0.09 (0.42) | 0.12 (0.41) | 0.75 (1.15) | 0.88 (1.13) | 0.12 (0.52) | <0.0001 | 0.0055 |
| Respiratory rate | 23.37 (5.91) | 23.27 (5.30) | 22.24 (5.41) | 24.47 (7.10) | 27.70 (6.86) | 0.0002 | 0.0034 |
| Glasgow Coma Scale | 15.00 (0.00) | 14.99 (0.11) | 11.18 (3.46) | 10.56 (4.01) | 14.89 (0.60) | <0.0001 | <0.0001 |
| Albumin | 3.16 (0.76) | 3.60 (0.66) | 3.66 (0.90) | 2.82 (0.81) | 2.54 (0.64) | <0.0001 | <0.0001 |
| Calcium | 8.51 (1.15) | 9.05 (0.93) | 8.82 (0.88) | 8.36 (1.18) | 7.74 (0.99) | <0.0001 | 0.0007 |
| Hemoglobin | 11.13 (2.15) | 12.65 (2.34) | 13.55 (1.70) | 11.00 (2.68) | 10.59 (2.29) | <0.0001 | <0.0001 |
| Sodium | 135.28 (4.86) | 136.75 (4.61) | 139.33 (6.22) | 136.86 (7.19) | 131.82 (4.73) | <0.0001 | <0.0001 |
| Chloride | 100.52 (5.96) | 98.92 (6.72) | 101.25 (7.72) | 102.61 (9.44) | 97.98 (7.52) | 0.0016 | 0.4073 |
| Potassium | 4.35 (0.94) | 4.20 (0.74) | 4.17 (0.90) | 4.39 (1.10) | 4.51 (0.91) | 0.2908 | 0.5837 |
| Creatinine | 2.67 (2.89) | 1.53 (1.13) | 1.62 (1.59) | 2.16 (1.77) | 2.99 (1.95) | 0.0001 | 0.058 |
| INR | 1.80 (1.40) | 1.26 (0.49) | 1.24 (0.25) | 1.66 (0.79) | 1.60 (0.61) | 0.0061 | 0.6697 |
| Platelets | 226.70 (150.64) | 271.02 (138.07) | 226.36 (96.93) | 246.57 (142.17) | 176.40 (132.56) | 0.0037 | 0.5978 |
| Total bilirubin | 1.03 (0.77) | 0.96 (1.18) | 1.20 (1.30) | 1.51 (2.13) | 3.04 (5.36) | <0.0001 | 0.037 |
| Lactate | 3.14 (2.01) | 4.62 (2.43) | 6.54 (3.93) | 5.06 (3.38) | 6.42 (3.06) | <0.0001 | 0.9998 |
| Bicarb | 21.45 (5.12) | 23.07 (6.63) | 22.41 (5.53) | 20.35 (6.26) | 17.34 (4.28) | <0.0001 | 0.0045 |
| Glucose | 134.21 (76.72) | 174.04 (120.79) | 219.71 (107.02) | 165.82 (130.01) | 158.14 (106.58) | 0.0059 | 0.1654 |
| WBC count | 15.92 (13.18) | 15.15 (8.63) | 12.97 (7.65) | 17.35 (14.39) | 15.01 (11.07) | 0.4654 | 0.9577 |
| Neutrophil % | 69.45 (25.43) | 77.78 (20.98) | 71.51 (19.62) | 72.34 (22.69) | 66.40 (23.03) | 0.0621 | 0.916 |
| Molecular markers, median (IQR) | | | | | | | |
| TNF | 28.00 (28.00-28.75) | 28.00 (14.91-28.00) | 28.00 (12.54-45.46) | 28.00 (28.00-67.05) | 33.05 (28.00-111.57) | 0.0153 | 0.6233 |
| IL6 | 238.73 (50.47-2574.93) | 79.28 (28.80-507.23) | 195.15 (111.57-265.88) | 1239.17 (94.82-16522.19) | 550.31 (365.93-4712.73) | 0.0304 | 0.7676 |
| IL10 | 12.85 (12.64-60.08) | 12.64 (10.41-28.91) | 37.44 (12.64-84.24) | 45.75 (12.64-357.06) | 32.04 (12.64-248.75) | 0.0153 | 0.9977 |
| Angiopoietin 2 | 7255.92 (4054.97-14134.53) | 3785.47 (2498.68-7340.03) | 4902.66 (2578.62-9949.23) | 9207.28 (4631.33-20739.29) | 17328.32 (7559.41-34804.27) | 0.0001 | 0.0279 |
| TMB | 4.86 (3.63-6.47) | 3.66 (3.14-4.89) | 3.53 (2.46-4.13) | 4.86 (3.46-6.94) | 6.64 (4.05-12.35) | 0.0002 | 0.0075 |
| vWF | 3936.23 (2332.21-5751.45) | 2648.03 (2045.32-3797.92) | 3361.27 (1728.55-5063.69) | 3512.57 (2713.93-6164.93) | 4673.28 (3370.11-6491.03) | 0.0023 | 0.3438 |

**Table S4:** Comparison of worst measurement for clinical variables and molecular markers across phenotypes at 24 hours in the derivation cohort. Phenotypes H1 and H2 were specifically compared against each other using Tukey tests because these were the two highest mortality groups

|  | L1  N=178 (28%) | L2  N=135 (21%) | M  N=102 (16%) | H1  N=125 (19%) | H2  N=102 (16%) | *P*-value | H1 vs H2 |
| --- | --- | --- | --- | --- | --- | --- | --- |
| Clustered clinical variables, mean±SD | | | | | | | |
| Temperature | 37.64 (1.26) | 37.64 (1.18) | 38.03 (1.43) | 36.87 (1.72) | 37.23 (1.35) | 0.6907 | 0.9515 |
| Heart rate | 102.15 (17.68) | 107.53 (21.04) | 112.15 (18.67) | 103.70 (26.80) | 116.33 (17.93) | <0.0001 | 0.0407 |
| Systolic BP | 82.56 (11.43) | 113.74 (24.79) | 107.14 (21.83) | 85.39 (15.11) | 84.04 (17.95) | <0.0001 | 0.1585 |
| Diastolic BP | 46.64 (8.87) | 65.03 (14.47) | 59.11 (14.55) | 45.20 (11.99) | 44.96 (10.84) | <0.0001 | 0.9916 |
| Cardiovascular score | 0.93 (1.39) | 0.15 (0.70) | 0.75 (1.41) | 1.70 (1.68) | 1.04 (1.45) | <0.0001 | 0.8103 |
| Respiratory score | 0.08 (0.31) | 0.15 (0.54) | 0.82 (1.01) | 0.93 (1.11) | 0.22 (0.59) | <0.0001 | 0.1151 |
| Respiratory rate | 23.67 (6.08) | 26.75 (8.33) | 25.12 (7.83) | 24.52 (8.66) | 26.80 (7.12) | 0.0009 | 0.0306 |
| Glasgow Coma Scale | 14.98 (0.19) | 14.92 (0.30) | 9.73 (4.19) | 9.83 (4.10) | 14.51 (1.60) | <0.0001 | <0.0001 |
| Albumin | 3.08 (0.77) | 3.62 (0.89) | 3.38 (0.75) | 2.42 (0.79) | 2.50 (0.75) | <0.0001 | 0.6994 |
| Calcium | 8.58 (1.04) | 9.02 (1.11) | 9.14 (1.02) | 8.00 (0.92) | 7.94 (0.94) | <0.0001 | 0.9999 |
| Hemoglobin | 11.36 (2.31) | 12.83 (2.71) | 13.30 (2.11) | 10.20 (2.17) | 9.89 (2.60) | <0.0001 | 0.8541 |
| Sodium | 135.64 (5.41) | 134.84 (6.32) | 139.23 (4.96) | 139.73 (8.45) | 132.85 (5.90) | <0.0001 | <0.0001 |
| Chloride | 101.04 (7.06) | 96.81 (8.22) | 101.75 (7.27) | 105.63 (9.40) | 99.51 (7.37) | <0.0001 | <0.0001 |
| Potassium | 4.11 (0.80) | 4.45 (1.05) | 4.22 (0.90) | 4.58 (1.19) | 4.65 (1.14) | <0.0001 | 0.6967 |
| Creatinine | 1.98 (1.67) | 2.54 (2.36) | 1.88 (1.46) | 3.02 (2.13) | 3.33 (2.52) | 0.0396 | 0.9921 |
| INR | 1.58 (1.91) | 1.97 (1.74) | 1.66 (1.04) | 2.05 (1.87) | 2.25 (1.61) | <0.0007 | 0.2344 |
| Platelets | 229.79 (129.77) | 230.45 (138.04) | 257.95 (137.86) | 232.51 (151.16) | 136.57 (108.28) | <0.0001 | <0.0001 |
| Total bilirubin | 1.33 (2.47) | 1.75 (2.59) | 1.04 (0.79) | 1.24 (2.62) | 3.25 (3.68) | <0.0001 | <0.0001 |
| Lactate | 3.09 (1.98) | 6.09 (2.78) | 5.91 (2.57) | 5.21 (3.78) | 7.56 (4.93) | <0.0001 | <0.0001 |
| Bicarb | 23.28 (4.25) | 19.45 (5.66) | 22.39 (6.24) | 19.69 (7.07) | 17.66 (4.26) | <0.0001 | 0.137 |
| Glucose | 140.25 (80.28) | 235.64 (210.49) | 217.87 (184.62) | 186.63 (170.34) | 128.94 (89.62) | <0.0001 | 0.2779 |
| WBC count | 14.85 (9.25) | 16.56 (9.54) | 16.36 (11.31) | 18.13 (14.70) | 18.55 (14.71) | 0.0117 | 0.9856 |
| Neutrophil % | 75.78 (20.23) | 73.17 (23.69) | 75.71 (21.81) | 73.69 (22.09) | 68.32 (25.87) | 0.0671 | 0.9689 |
| Molecular markers, median (IQR) | | | | | | | |
| TNF | 28.00 (17.41-32.61) | 28.00 (14.73-30.48) | 28.00 (16.67-35.01) | 28.00 (20.49-32.74) | 28.00 (26.54-137.65) | 0.069 | 0.9875 |
| IL6 | 164.80 (40.08-1129.91) | 147.25 (33.96-1766.51) | 457.52 (81.42-3842.01) | 342.65 (68.06-1787.35) | 1213.25 (164.53-13702.16) | 0.7662 | 0.8671 |
| IL10 | 15.85 (12.64-47.56) | 26.52 (12.64-97.40) | 21.64 (12.64-98.08) | 30.05 (12.64-120.94) | 40.66 (12.64-312.22) | 0.7627 | 0.7903 |
| Angiopoietin 2 | 6219.72 (3124.37-10875.65) | 8095.15 (3217.55-17075.40) | 6043.98 (3800.03-13005.64) | 9916.65 (4746.37-19064.21) | 24684.96 (9613.80-38689.72) | 0.493 | 0.9961 |
| TMB | 4.23 (3.17-5.55) | 4.29 (2.98-6.66) | 3.98 (3.39-6.10) | 5.31 (3.52-8.33) | 6.88 (4.94-10.51) | 0.3287 | 0.9898 |
| vWF | 3303.09 (2360.85-4708.34) | 3474.97 (2364.82-5421.20) | 3743.15 (2271.03-5215.95) | 3851.78 (2599.35-5903.59) | 5825.26 (4455.40-8097.86) | 0.0365 | 0.854 |

**Table S8:** Comparison of worst measurement for clinical variables and molecular markers across phenotypes at 24 hours in the validation cohort. Phenotypes H1 and H2 were specifically compared against each other using Tukey tests because these were the two highest mortality groups

|  | L1  N=109 (29%) | L2  N=97 (25%) | M  N=25 (7%) | H1  N=100 (26%) | H2  N=50 (13%) | *P*-value | H1 vs H2 |
| --- | --- | --- | --- | --- | --- | --- | --- |
| Clustered clinical variables, mean±SD | | | | | | | |
| Temperature | 37.31 (1.09) | 37.33 (1.02) | 38.26 (1.40) | 37.13 (1.18) | 36.94 (1.08) | 0.0011 | 0.0122 |
| Heart rate | 102.29 (18.48) | 106.45 (19.74) | 119.72 (17.38) | 102.96 (21.48) | 113.72 (17.63) | 0.0019 | 0.9986 |
| Systolic BP | 82.25 (12.39) | 104.18 (18.66) | 115.72 (21.17) | 93.24 (19.01) | 90.44 (13.89) | <0.0001 | <0.0001 |
| Diastolic BP | 45.52 (7.93) | 58.26 (11.05) | 65.60 (13.87) | 49.56 (11.05) | 54.20 (10.48) | <0.0001 | 0.0148 |
| Cardiovascular score | 1.66 (1.61) | 0.35 (0.98) | 0.84 (1.43) | 1.63 (1.60) | 1.18 (1.59) | <0.0001 | 0.0409 |
| Respiratory score | 0.09 (0.42) | 0.12 (0.41) | 0.75 (1.15) | 0.88 (1.13) | 0.12 (0.52) | <0.0001 | 0.9526 |
| Respiratory rate | 23.37 (5.91) | 23.27 (5.30) | 22.24 (5.41) | 24.47 (7.10) | 27.70 (6.86) | 0.0002 | 0.0012 |
| Glasgow Coma Scale | 15.00 (0.00) | 14.99 (0.11) | 11.18 (3.46) | 10.56 (4.01) | 14.89 (0.60) | <0.0001 | 0.0007 |
| Albumin | 3.16 (0.76) | 3.60 (0.66) | 3.66 (0.90) | 2.82 (0.81) | 2.54 (0.64) | <0.0001 | 0.0001 |
| Calcium | 8.51 (1.15) | 9.05 (0.93) | 8.82 (0.88) | 8.36 (1.18) | 7.74 (0.99) | <0.0001 | 0.0458 |
| Hemoglobin | 11.13 (2.15) | 12.65 (2.34) | 13.55 (1.70) | 11.00 (2.68) | 10.59 (2.29) | <0.0001 | 0.0001 |
| Sodium | 135.28 (4.86) | 136.75 (4.61) | 139.33 (6.22) | 136.86 (7.19) | 131.82 (4.73) | <0.0001 | <0.0001 |
| Chloride | 100.52 (5.96) | 98.92 (6.72) | 101.25 (7.72) | 102.61 (9.44) | 97.98 (7.52) | <0.0001 | 0.0014 |
| Potassium | 4.35 (0.94) | 4.20 (0.74) | 4.17 (0.90) | 4.39 (1.10) | 4.51 (0.91) | 0.1814 | 0.645 |
| Creatinine | 2.67 (2.89) | 1.53 (1.13) | 1.62 (1.59) | 2.16 (1.77) | 2.99 (1.95) | <0.0001 | 0.0376 |
| INR | 1.80 (1.40) | 1.26 (0.49) | 1.24 (0.25) | 1.66 (0.79) | 1.60 (0.61) | 0.0004 | 0.394 |
| Platelets | 226.70 (150.64) | 271.02 (138.07) | 226.36 (96.93) | 246.57 (142.17) | 176.40 (132.56) | 0.0043 | 0.9822 |
| Total bilirubin | 1.03 (0.77) | 0.96 (1.18) | 1.20 (1.30) | 1.51 (2.13) | 3.04 (5.36) | <0.0001 | 0.0064 |
| Lactate | 3.14 (2.01) | 4.62 (2.43) | 6.54 (3.93) | 5.06 (3.38) | 6.42 (3.06) | <0.0001 | 0.9705 |
| Bicarb | 21.45 (5.12) | 23.07 (6.63) | 22.41 (5.53) | 20.35 (6.26) | 17.34 (4.28) | <0.0001 | 0.0075 |
| Glucose | 134.21 (76.72) | 174.04 (120.79) | 219.71 (107.02) | 165.82 (130.01) | 158.14 (106.58) | 0.0151 | 0.1214 |
| WBC count | 15.92 (13.18) | 15.15 (8.63) | 12.97 (7.65) | 17.35 (14.39) | 15.01 (11.07) | 0.2356 | 0.6885 |
| Neutrophil % | 69.45 (25.43) | 77.78 (20.98) | 71.51 (19.62) | 72.34 (22.69) | 66.40 (23.03) | 0.1215 | 0.965 |
| Molecular markers, median (IQR) | | | | | | | |
| TNF | 28.00 (28.00-28.75) | 28.00 (14.91-28.00) | 28.00 (12.54-45.46) | 28.00 (28.00-67.05) | 33.05 (28.00-111.57) | 0.7408 | 0.9312 |
| IL6 | 238.73 (50.47-2574.93) | 79.28 (28.80-507.23) | 195.15 (111.57-265.88) | 1239.17 (94.82-16522.19) | 550.31 (365.93-4712.73) | 0.7397 | 0.9979 |
| IL10 | 12.85 (12.64-60.08) | 12.64 (10.41-28.91) | 37.44 (12.64-84.24) | 45.75 (12.64-357.06) | 32.04 (12.64-248.75) | 0.9444 | 0.9997 |
| Angiopoietin 2 | 7255.92 (4054.97-14134.53) | 3785.47 (2498.68-7340.03) | 4902.66 (2578.62-9949.23) | 9207.28 (4631.33-20739.29) | 17328.32 (7559.41-34804.27) | 0.1778 | 0.8485 |
| TMB | 4.86 (3.63-6.47) | 3.66 (3.14-4.89) | 3.53 (2.46-4.13) | 4.86 (3.46-6.94) | 6.64 (4.05-12.35) | 0.4151 | 0.7503 |
| vWF | 3936.23 (2332.21-5751.45) | 2648.03 (2045.32-3797.92) | 3361.27 (1728.55-5063.69) | 3512.57 (2713.93-6164.93) | 4673.28 (3370.11-6491.03) | 0.2794 | 0.531 |

**Table S9**: Intercepts and coefficients of the multinomial model. Type M was used as the reference group.

|  | **H1** | **H2** | **L1** | **L2** |
| --- | --- | --- | --- | --- |
| **(Intercept)** | 41.3 | 41.0 | -48.9 | -35.2 |
| **Albumin** | -0.833 | -1.00 | -0.806 | -0.121 |
| **Bicarb** | -0.148 | -0.0782 | 0.160 | -0.0548 |
| **Calcium** | -1.46 | -1.04 | -0.366 | 0.102 |
| **Chloride** | 0.0149 | 0.0349 | 0.102 | -0.0359 |
| **Diastolic.BP** | -0.0737 | -0.0891 | -0.0439 | 0.0497 |
| **Glasgow.Coma.Scale** | 0.135 | 1.30 | 5.29 | 4.45 |
| **Heart.Rate** | -0.0222 | 0.0233 | -0.0275 | -0.0209 |
| **Hemoglobin** | -0.605 | -0.750 | -0.456 | -0.335 |
| **Lactate** | -0.150 | 0.248 | -0.277 | 0.113 |
| **Sodium** | 0.0775 | -0.110 | -0.0865 | -0.122 |
| **Systolic.BP** | -0.0743 | -0.0823 | -0.0959 | 0.0121 |
| **Temperature** | -0.415 | -0.442 | -0.0582 | -0.147 |

**Table S10:** Multinomial model accuracy in the derivation cohort

| **Predicted** | **Actual phenotype** | | | | | **Predicted total** | **High risk**  **mis-predicted** |
| --- | --- | --- | --- | --- | --- | --- | --- |
|  | **L1** | **L2** | **M** | **H1** | **H2** |  |  |
| **l1** | 158 (89%) | 10 (7%) | 0 (0%) | 2 (2%) | 22 (22%) | 192 | 0.125 |
| **l2** | 7 (4%) | 119 (88%) | 4 (4%) | 0 (0%) | 4 (4%) | 134 | 0.029851 |
| **m** | 0 (0%) | 2 (1%) | 88 (86%) | 5 (4%) | 1 (1%) | 96 | 0.0625 |
| **h1** | 1 (1%) | 1 (1%) | 9 (9%) | 104 (83%) | 6 (6%) | 121 |  |
| **h2** | 12 (7%) | 3 (2%) | 1 (1%) | 14 (11%) | 69 (68%) | 99 |  |
| **Actual total** | 178 | 135 | 102 | 125 | 102 |  |  |

**Table S11: Comparison of high-risk types in ProCESS**

|  | **H1** | **H2** | **Delta (SENECA type applied to ProCESS)** |
| --- | --- | --- | --- |
| **Percentage of cohort** | 19 | 16 | 16 |
| **Notable organ dysfunctions** | CV, pulm, neuro, renal | Hepatic, heme, renal | CV, other, heme, hepatic, inflamm, pulm, renal |
| **28 d mortality %** | 34.4 | 41.2 | 44 |
| **60 d mortality %** | 42.4 | 44.1 |  |
| **365 d mortality %** | 55.2 | 52.9 | 57 |
| **Mean age** | 65.9 | 56.7 | 63 |
| **SBP** | 85.39 (15.11) | 84.04 | 90 |
| **Lactate** | 5.21 (3.78) | 7.56 | 6.9 |
| **Creatinine** | 3.02 (2.13) | 3.33 | 2.3 |
| **Bili** | 1.24 (2.62) | 3.25 | 1.4 |
| **Hemoglobin** | 10.20 | 9.89 | 12 |
| **Platelets** | 232.51 (151.16) | 136.57 | 175 |
| **GCS** | 9.83 (4.10) | 14.51 | 10.9 |
| **IL6, median** | 343 [68 – 1,787] | 1213 [164 – 13,702] | 1091 [180 – 8,919] |
| **IL10** | 30 [13 – 121] | 41 [13 – 312] | 49 [18 – 237] |

**Table S12a: Pressor requirement of phenotypes over time in derivation cohort**

| **Pressors required** | L1 (N=178) | L2 (N=135) | M (N=102) | H1 (N=125) | H2 (N=102) |
| --- | --- | --- | --- | --- | --- |
| **0 Hours** | 51 (29%) | 6 (4%) | 23 (22%) | 63 (50%) | 31 (30%) |
| **6 Hours** | 95 (53%) | 28 (21%) | 36 (35%) | 89 (71%) | 62 (61%) |
| **24 Hours** | 62 (35%) | 27 (20%) | 30 (29%) | 63 (50%) | 55 (54%) |
| **72 Hours** | 17 (10%) | 8 (6%) | 9 (9%) | 19 (15%) | 24 (24%) |

**Table S12b: Pressor requirement of phenotypes over time in validation cohort**

| **Pressors required** | L1 (N=109) | L2 (N=97) | M (N=25) | H1 (N=100) | H2 (N=50) |
| --- | --- | --- | --- | --- | --- |
| **0 Hours** | 55 (50%) | 11 (11%) | 7 (28%) | 53 (53%) | 18 (36%) |
| **6 Hours** | 70 (64%) | 21 (22%) | 9 (36%) | 75 (75%) | 33 (66%) |
| **24 Hours** | 33 (30%) | 8 (8%) | 6 (24%) | 41 (41%) | 27 (54%) |
| **72 Hours** | 14 (13%) | 6 (6%) | 1 (4%) | 12 (12%) | 8 (16%) |

# Supplementary Figures

#
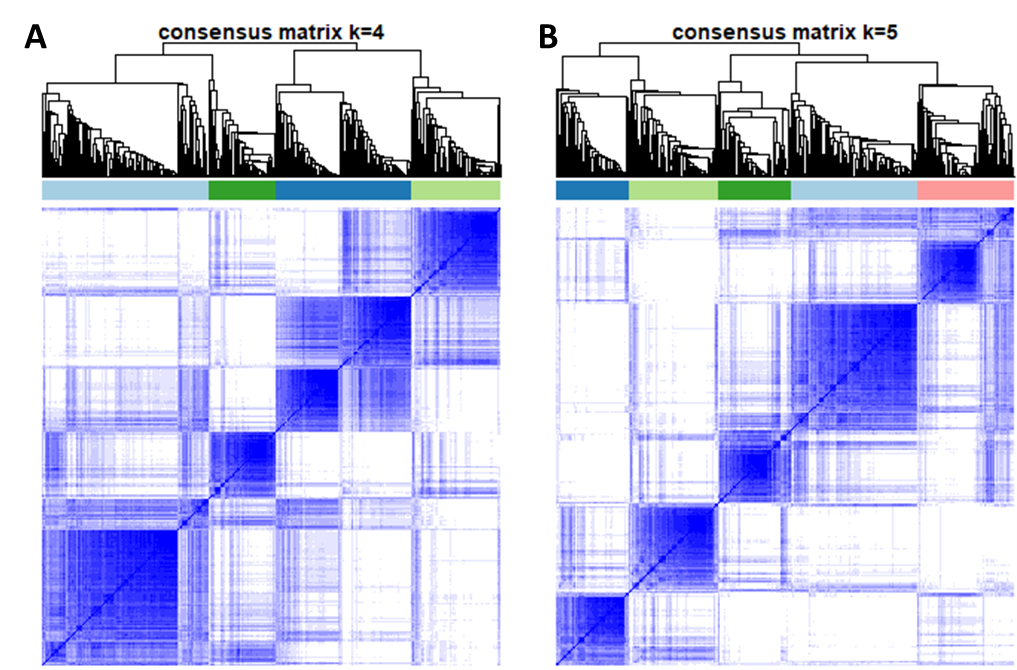


**Figure S1:** Consensus plots for 4 (A) and 5 (B) clusters using hierarchical clustering


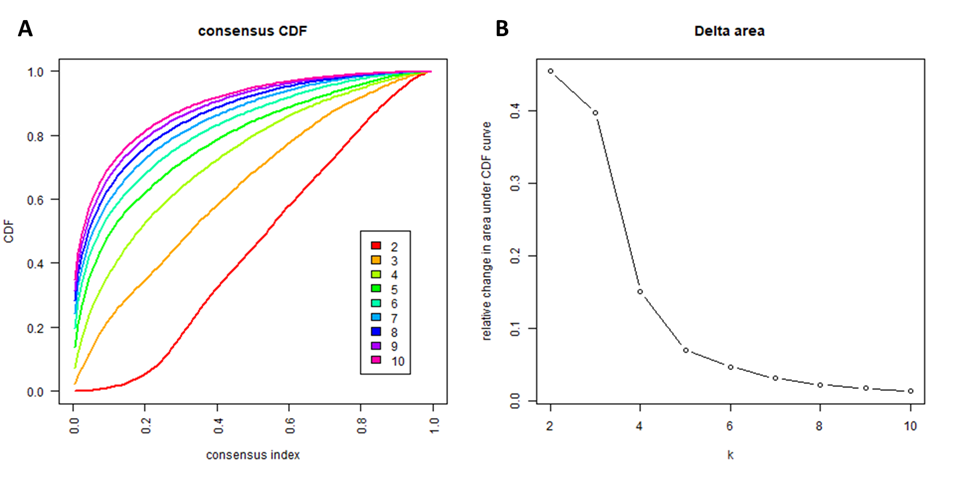


**Figure S2:** Cumulative distribution function for varying numbers of clusters using hierarchical clustering


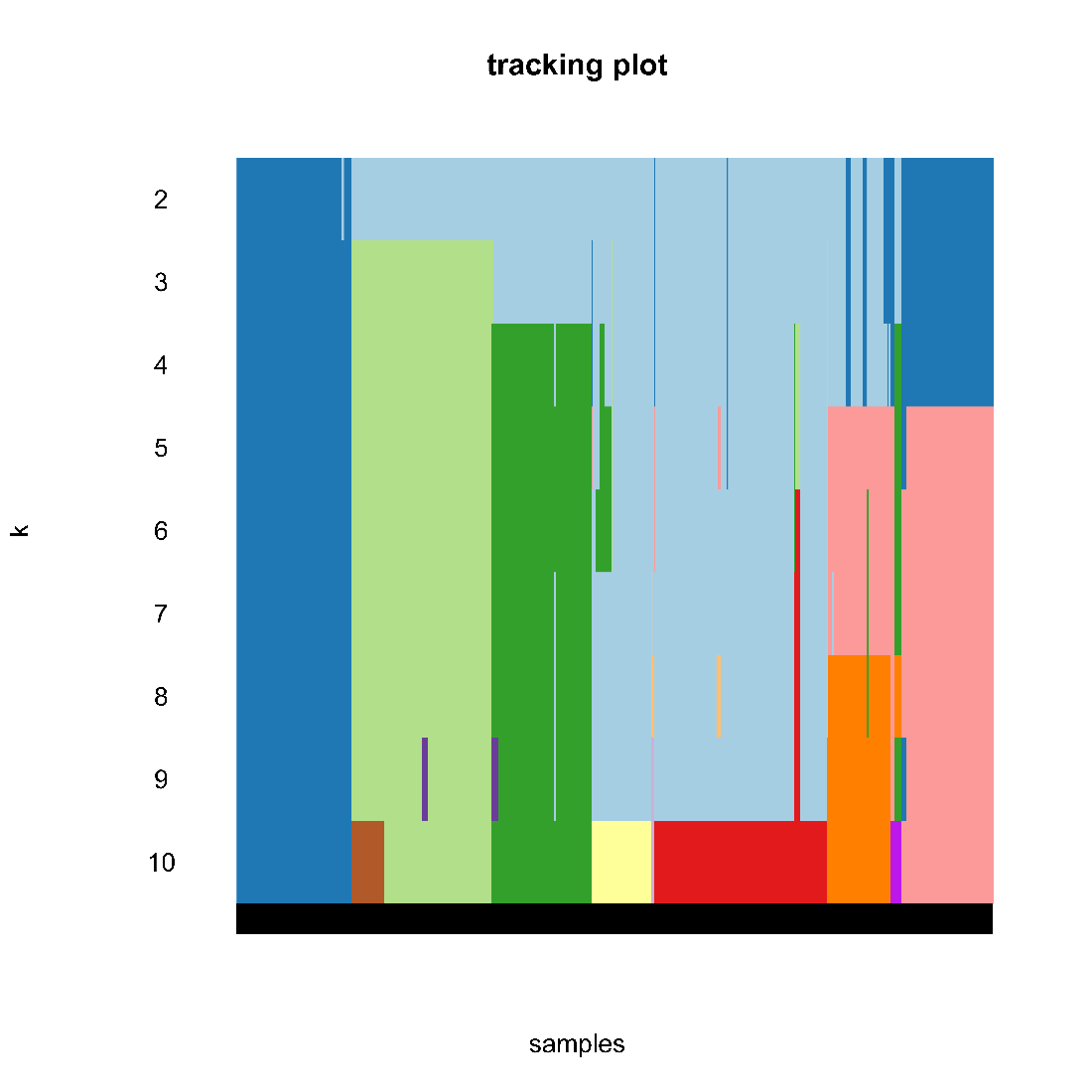


**Figure S3:** Cluster membership tracking plot using hierarchical clustering


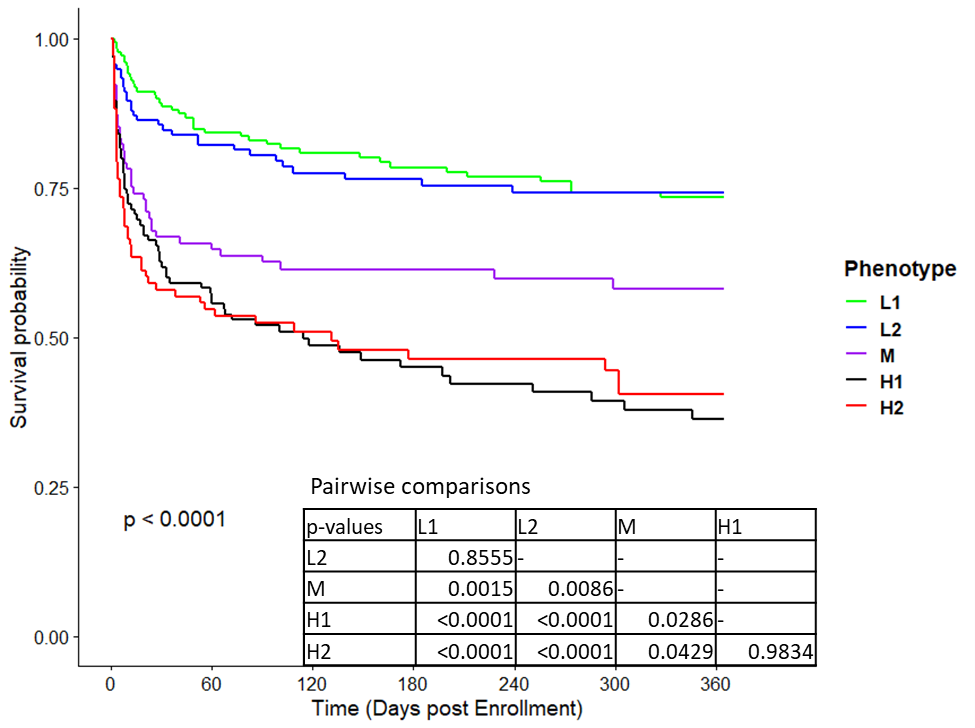


**Figure S4:** 365-day Kaplan-Meier Curves for derivation cohort


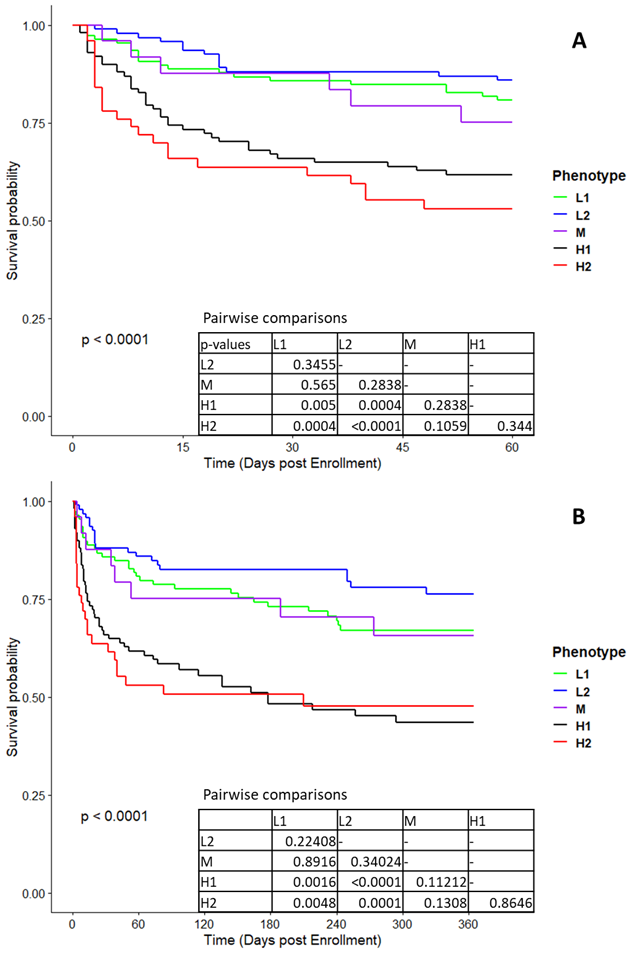


**Figure S5:** 60-day (A) and 365-day (B) Kaplan-Meier Curves for validation cohort


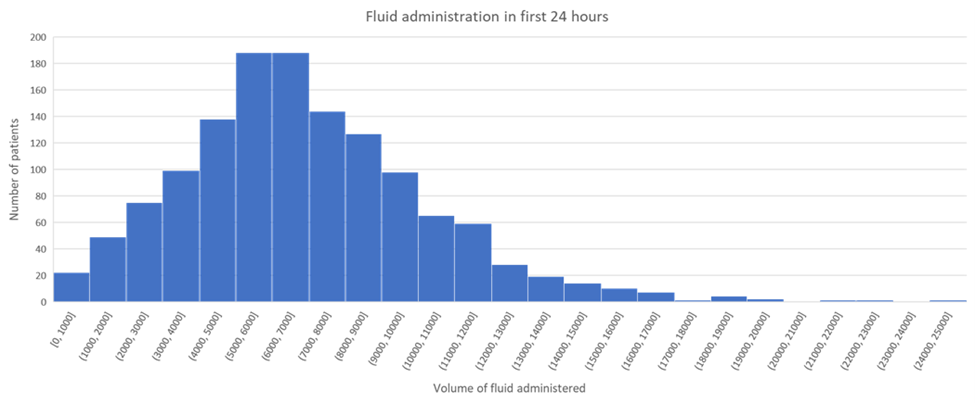


**Figure S6:** Histogram of fluid administration in the first 24 hours. Y axis indicates number of patients receiving the range of fluid indicated on the x-axis

**
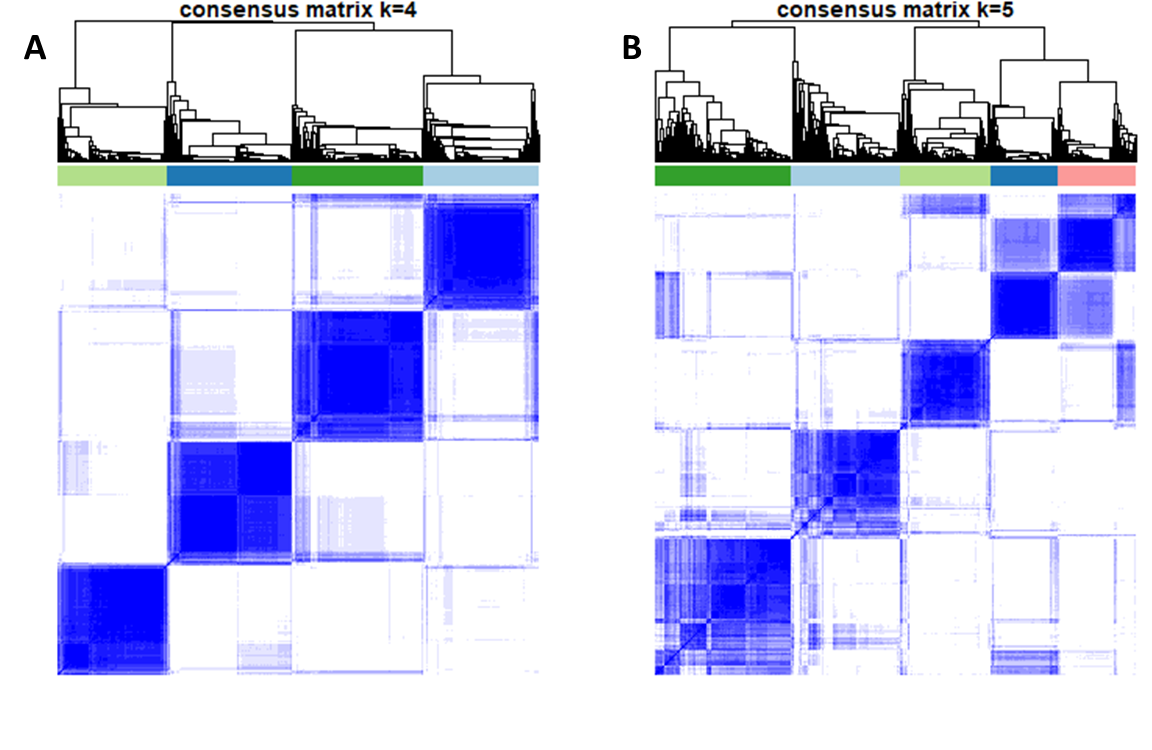
**

**Figure S7:** Consensus plots for 4 (A) and 5 (B) clusters using K-means clustering


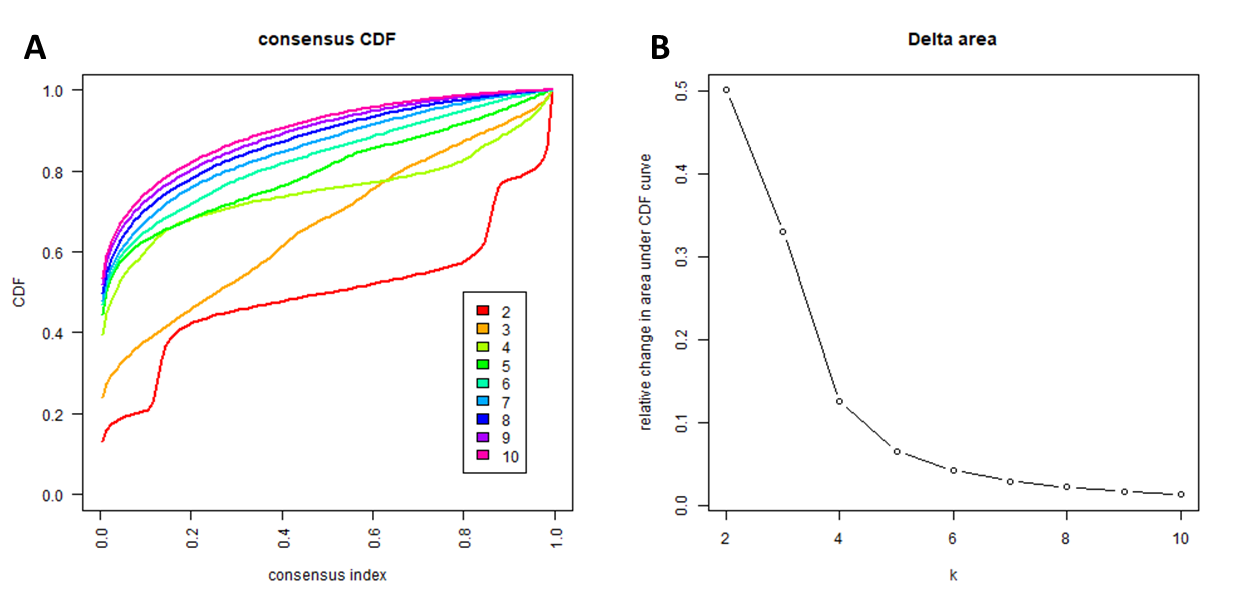
**Figure S8:** Cumulative distribution function for varying numbers of clusters using K-means clustering

**
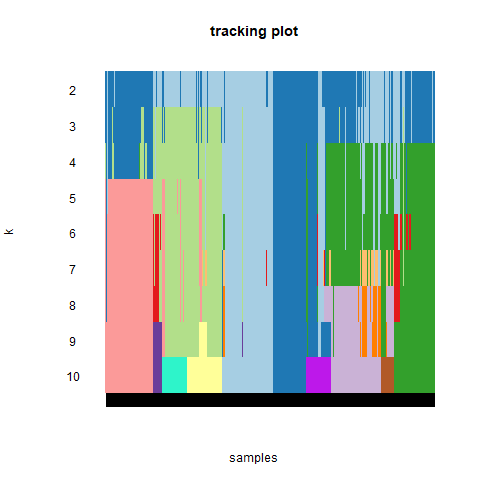
**

**Figure S9:** Cluster membership tracking plot using K-means clustering

**
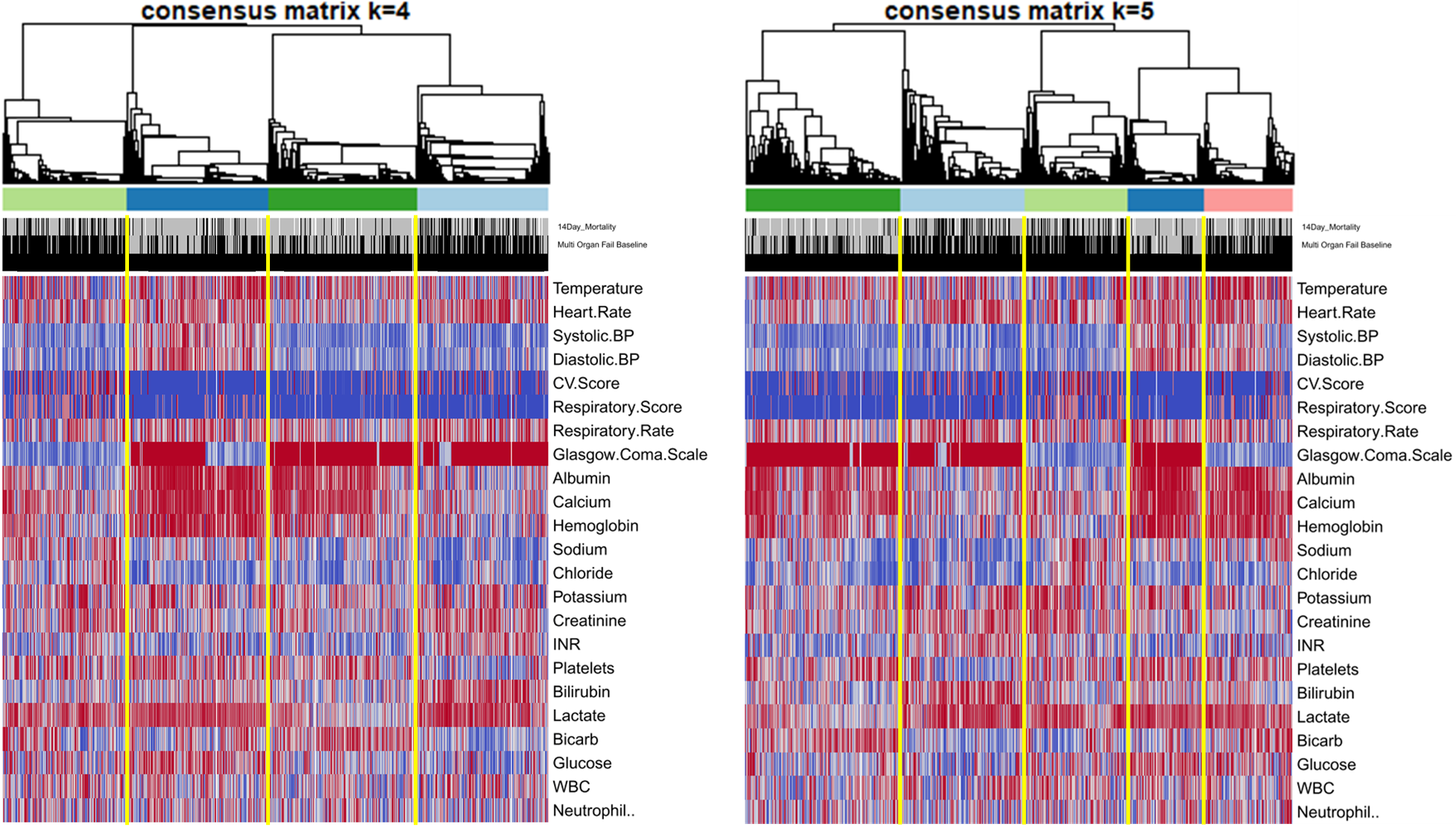
**

**Figure S10:** Heatmaps for K-means clustering with 4 and 5 clusters. Compared with figure 1, where patterns are more easily recognizable, similar clusters are seen with 5 clusters but less distinctively, especially for moderate and high risk groups.
